# Azithromycin Treatment Alters Gene Expression in Inflammatory, Lipid Metabolism, and Cell Cycle Pathways in Well-Differentiated Human Airway Epithelia

**DOI:** 10.1371/journal.pone.0005806

**Published:** 2009-06-05

**Authors:** Carla Maria P. Ribeiro, Harry Hurd, Yichao Wu, Mary E. B. Martino, Lisa Jones, Brian Brighton, Richard C. Boucher, Wanda K. O'Neal

**Affiliations:** 1 Cystic Fibrosis Center, The University of North Carolina at Chapel Hill, Chapel Hill, North Carolina, United State of America; 2 Department of Medicine, The University of North Carolina at Chapel Hill, Chapel Hill, North Carolina, United State of America; 3 Department of Statistics, The University of North Carolina at Chapel Hill, Chapel Hill, North Carolina, United State of America; LMU University of Munich, Germany

## Abstract

Prolonged macrolide antibiotic therapy at low doses improves clinical outcome in patients affected with diffuse panbronchiolitis and cystic fibrosis. Consensus is building that the therapeutic effects are due to anti-inflammatory, rather than anti-microbial activities, but the mode of action is likely complex. To gain insights into how the macrolide azithromycin (AZT) modulates inflammatory responses in airways, well-differentiated primary cultures of human airway epithelia were exposed to AZT alone, an inflammatory stimulus consisting of soluble factors from cystic fibrosis airways, or AZT followed by the inflammatory stimulus. RNA microarrays were conducted to identify global and specific gene expression changes. Analysis of gene expression changes revealed that the AZT treatment alone altered the gene profile of the cells, primarily by significantly increasing the expression of lipid/cholesterol genes and decreasing the expression of cell cycle/mitosis genes. The increase in cholesterol biosynthetic genes was confirmed by increased filipin staining, an index of free cholesterol, after AZT treatment. AZT also affected genes with inflammatory annotations, but the effect was variable (both up- and down-regulation) and gene specific. AZT pretreatment prevented the up-regulation of some genes, such as MUC5AC and MMP9, triggered by the inflammatory stimulus, but the up-regulation of other inflammatory genes, e.g., cytokines and chemokines, such as interleukin-8, was not affected. On the other hand, HLA genes were increased by AZT. Notably, secreted IL-8 protein levels did not reflect mRNA levels, and were, in fact, higher after AZT pretreatment in cultures exposed to the inflammatory stimulus, suggesting that AZT can affect inflammatory pathways other than by altering gene expression. These findings suggest that the specific effects of AZT on inflamed and non-inflamed airway epithelia are likely relevant to its clinical activity, and their apparent complexity may help explain the diverse immunomodulatory roles of macrolides.

## Introduction

Prolonged macrolide antibiotic therapy at low doses improves clinical outcome in patients affected with diffuse panbronchiolitis (DPB) and cystic fibrosis (CF), lethal conditions characterized by chronic pulmonary Pseudomonas aeruginosa infections, diffuse inflammation, and airways obstruction [1], [2]. These drugs are also being used to treat patients with other inflammatory lung conditions, such as sinusitis, chronic obstructive pulmonary disease, asthma, and bronchiectasis [3]. Both antimicrobial [4], [5] and immunomodulatory activities [6]–[17] are associated with macrolide treatment. While antimicrobial activity is one aspect likely contributing to the clinical outcome, the immunomodulatory effects are becoming increasingly recognized as critical to the mode of action of these drugs for the treatment of pulmonary diseases as well as inflammatory diseases in other organs, such as inflammatory bowel disease, arthritis, and psoriasis [3].

Whereas the antimicrobial activity of macrolides is known to result primarily from inhibition of RNA-dependent protein synthesis in susceptible microorganisms [18], the mechanism of action concerning immunomodulation is not entirely clear. For example, macrolide treatment was shown to decrease the levels of interleukin 8 (IL-8) and/or IL-1β in bronchoalveolar lavage fluid from DPB and bronchiolitis obliterans patients [6]. Other studies have reported reduced levels of pro-inflammatory cytokines triggered by P. aeruginosa challenges or loss of CFTR in murine lungs [7], reduced production of cytokines by airway epithelia [8]–[10], reduced neutrophil influx, chemotaxis and number [7], [8], [11]–[13], inhibition of airway epithelia-mediated neutrophil survival [14], and increased phagocytosis of apoptotic bronchial epithelial cells by alveolar macrophages [17]. At a gene expression level, macrolides have been reported to reduce induction of mucin 5AC (MUC5AC) synthesis after inflammatory stimuli [19], [20] and to decrease the expression of matrix metallopeptidase 9 (MMP9) [21], [22]. The understanding of macrolide action is complicated by other findings suggesting that the macrolide azithromycin (AZT) may actually exhibit some pro-inflammatory features, such as acute stimulation of neutrophil degranulation and enzyme release, increased phagocytosis-associated oxidative burst, and increased serum IL-1β [16].

To gain new insights into the mode of action of AZT in airways, we evaluated its effect on gene expression in well-differentiated primary human bronchial epithelial (HBE) cultures using RNA expression microarrays and measurements of secreted IL-8. Based upon published reports of immunomodulatory properties, we hypothesized that AZT would blunt the pro-inflammatory response induced by exposure of HBE cultures to a potent inflammatory stimulus, supernatant of mucopurulent material (SMM) from CF airways [23], [24]. Our findings highlight the complex nature of the AZT effect on airway cells, which involves up-regulation of lipid/cholesterol biosynthetic pathways, suppression of cell division pathways, and specific modulation of inflammatory genes, including down-regulation of mucin production. Additionally, we demonstrate that mRNA levels of IL-8 are not correlated to secreted protein levels, adding additional complexity to the effect of AZT on the modulation of inflammatory responses.

## Methods

### Cell culture

Human bronchial epithelial (HBE) cells were provided by the Tissue Core of the University of North Carolina CF Center under the auspices of protocols approved by the Institutional Committee on the Protection of the Rights of Human Subjects. Cells were harvested by enzymatic digestion from portions of main stem or lumbar bronchi, cultured, and studied as well-differentiated primary cultures maintained at an air-liquid surface interface as previously described [23], [24].

### Supernatant of mucopurulent material (SMM) from CF airways

Mucopurulent material was harvested from the airway lumens of excised human CF lungs infected with *Pseudomonas aeruginosa* at the time of transplant and provided by the Tissue Core of the University of North Carolina CF Center as previously described [23], [24]. This material was centrifuged at 100,000 rpm (60 min, 4°C), the supernatant filtered through a 0.2 µm filter and frozen at −80°C. To induce airway epithelial inflammation, the mucosal surfaces of well-differentiated HBE cultures were exposed to 30 µl SMM for the indicated times in the Figures. 30 µl PBS were used as a control for SMM as previously reported [23], [24].

### Azithromycin treatment

AZT (Pfizer) was freshly added (1∶1000 dilution from frozen stocks kept in −80°C) daily and continuously applied to the serosal compartment, in the absence or presence of mucosal exposure to 30 µl PBS or SMM, throughout the course of the studies. The concentrations of AZT used in this study are in agreement with previous pharmacokinetic studies of long-term administration of AZT (500 mg per day) in CF patients showing that the concentration of AZT in sputum ranged from 12 to 53 µg/ml and was still detectable at concentrations in the range of 4 to 27 µg/ml 10 days after the last dose [25].

### RNA microarrays

The microarray experiments were performed according to the MIAME guidelines, and the data from the experiments were deposited in a publicly accessible database (ftp://ftp.ncbi.nih.gov/pub/geo/DATA/supplementary/series/; series record GSE10592). The experimental protocol for these studies is depicted in Table 1. RNA harvested at different time points from 4 individual codes of HBE treated with PBS, SMM or AZT, or pretreated with AZT prior to mucosal exposure to SMM, was hybridized to human Affymetrix U133 Plus 2.0 arrays. Hybridizations were conducted according to standard protocols by the University of North Carolina Genomics Analysis Core. Details of the statistical analyses are found in Supporting Information (Methods S1). Briefly, expression levels were estimated using GCRMA [26]. The local-pooled-error method (LPE) was used for comparisons of selected treatment pairs and the z-score produced by LPE was used for selecting probesets with significantly different gene expression. Probesets ranked by z-scores were used to determine gene ontology (GO) groups that were over-represented in the differentially regulated gene lists using hypergeometric probabilities, using a method specifically developed by Harry Hurd (see Supporting Information, Methods S1, for further documentation). Hypergeometric probabilities for each of the significant gene ontology classifications listed for the top probesets up-regulated or down-regulated by the various treatments are shown in Multi-Gene Ontology Matrix (MGM) Tables (Supporting Information, Table S6). Hierarchical clustering was used as a complementary method. Clustering was limited to only differentially expressed genes as described in the Supporting Information (Methods S1). Further analysis of the data was carried out using Ingenuity Pathways Analysis™ software available through a license to the University of North Carolina Lineberger Cancer Center (Ingenuity Systems, Inc., Redwood City, CA). When the software is utilized, the specific parameters used for the network visualized are presented in the figure legend.

**Table 1:** Experimental conditions utilized in the microarray studies.

Treatment Name (N)	Treatment Description
T0 (3)	No treatment, time 0 control
PBS 6 hr (4)	PBS-exposed, harvested at 6 hr
PBS 24 hr (4)	PBS-exposed, harvested at 24 hr
AZT 6 hr (3)	AZT-treated, harvested at 6 hr
AZT 24 hr (4)	AZT-treated, harvested at 24 hr
AZT 48 hr (3)	AZT-treated, harvested at 48 hr
SMM 6 hr (4)	SMM-exposed, harvested at 6 hr
SMM 24 hr (3)	SMM-exposed, harvested at 24 hr
AZT 48 hr+SMM 6 hr (3)	AZT-treated for 48 hr; SMM-exposed during last 6 hrs
AZT 72 hr+SMM 24 hr (3)	AZT-treated for 72 hr; SMM-exposed during last 24 hrs

PBS: 30 µl mucosal phosphate-buffered saline; AZT: 30 µg/ml serosal azithromycin. SMM: 30 µl mucosal supernatant from mucopurulent material from CF airways. N = number of arrays from 4 different human HBE codes that were used for subsequent analyzes.

### Mevastatin treatment

Mevastatin (Fluka) was used to inhibit the activity of 3-hydroxy-3-methylglutaryl-CoA reductase, which mediates cholesterol biosynthesis. Mevastatin was serosally added daily at a concentration of 50 µM throughout the course of the tudies, in the absence or presence of mucosal exposure to 30 µl PBS or SMM, and/or serosal exposure to 30 µg/ml AZT.

### MUC5AC staining

Immunocytochemistry of MUC5AC was performed according to standard techniques, utilizing a mouse monoclonal primary antibody (1∶1,000 dilution; Abcam) and an immunoperoxidase detection system, which contains mouse IgG (VECTASTAIN, Vector Laboratories).

### Real-time quantitative RT-PCR

200 ng of the RNA from HBE cultures treated as described in the figure legends was reverse transcribed using Superscript II reverse transcriptase (Invitrogen). Quantitative real-time PCR was performed using 100 ng of cDNA, Taqman primers and a FAM labeled Taqman probe for *MUC5AC* (Hs01365601_m1) and *SREBF* (Hs01088691_m1) (Applied Biosystems) on a 7500 Fast Real-Time PCR system (Applied Biosystems). Data were normalized to 18S expression according to the method of Pfaff [27].

### IL-8 secretion

IL-8 secretion into the serosal media was assessed at different time points in cultures treated with vehicle or AZT alone, or mucosally exposed to 30 µl PBS or SMM following the AZT pretreatment. The serosal media were freshly added and collected daily for IL-8 determination. IL-8 was measured by ELISA (R&D Systems) in duplicate as previously described [24].

### Filipin staining

Filipin staining was performed according to a slight modification of the method of White et al [28]. Briefly, following the different treatments, HBE cultures were serosally and mucosally rinsed three times with PBS, fixed with 4% paraformaldehyde as previously reported [23], [24], rinsed three times with PBS and incubated with 0.05 mg/ml filipin (Sigma) in PBS for 60 min on a shaker in the dark. Cultures were rinsed in PBS and filipin fluorescence intensity studied by laser confocal microscopy in the X-Y scanning mode (Leica, model TCS 4D; PL APO 63×/1.20 mm water lens) and quantified as reported in previous studies [23], [24], [29].

### Statistical analysis

Data in bar graphs represent the mean±SEM from at least 3 experiments. Where appropriate, data were analyzed by two-way analysis of variance (ANOVA) and statistical significance was defined with p<0.05.

## Results

### Microarray studies

Microarray experiments were performed to evaluate global effects of AZT, with and without inflammatory challenge, on gene expression in the well-differentiated HBE cultures. RNA was harvested from cultures at various times after designated treatments and hybridized to the Affymetrix U133 Plus 2.0 microarrays. The specific treatments utilized in this study are depicted in Table 1. Although not all possible PBS controls were included in the study (e.g., 72 hr PBS), it was recognized that gene expression could change across time and culture conditions. Therefore, we were careful to do direct comparisons of treatments in subsequent analyses only between treatments harvested under the same culture conditions and culture times. Several different methods were used to comprehend and interpret the gene expression responses.

### The number of differentially-regulated probesets reveals AZT-dependent alterations in gene expression

Table 2 summarizes the number of differentially expressed probesets for the key comparisons as defined by the LPE z-score <0.05. Using this criterion, AZT alone at 24 hr exposure led to differential expression of 468 genes, with a fairly equal representation of up- versus down-regulation, demonstrating the ability of AZT to alter cellular gene expression in the absence of any additional treatment. As expected, SMM, which includes a complex mixture of stimulatory infectious and inflammatory factors, led to the up-regulation of a large number of mRNA sequences, with fewer down-regulated probesets, at both 6 and 24 hrs. Importantly, when gene expression was compared between SMM-exposed cultures with or without AZT pretreatment (last two rows of Table 2), a large number of gene expression changes could be identified (649 for 6 hr SMM exposure and 475 for 24 hr SMM exposure). These results demonstrated that AZT pretreatment significantly altered the way in which HBE responded to the inflammatory SMM stimulus. AZT affected gene expression after SMM treatment in both directions, with down-regulation being the stronger trend. The complete lists of differentially regulated genes, as defined by our criteria, are provided in Supporting Information, Table S2.

**Table 2:** Number of differentially expressed probesets in selected paired comparisons.

Pair Comparison	Up-regulated	Down-regulated	Total
AZT 24 hr vs PBS 24 hr	238	230	468
SMM 6 hr vs PBS 6 hr	638	110	748
SMM 24 hr vs PBS 24 hr	562	221	783
AZT 48 hr (+SMM 6 hr) vs. SMM 6 hr	303	346	649
AZT 72 hr (+SMM 24 hr) vs. SMM 24 hr	183	292	475

Table represents the number of significant probesets with LPE z.pv<0.05. A list of all differentially-regulated probesets is found in Supporting Information, Table S2.

### Hierarchical clustering reveals complexity among the AZT effects in the presence and absence of inflammatory stimulus

**Figure 1:**
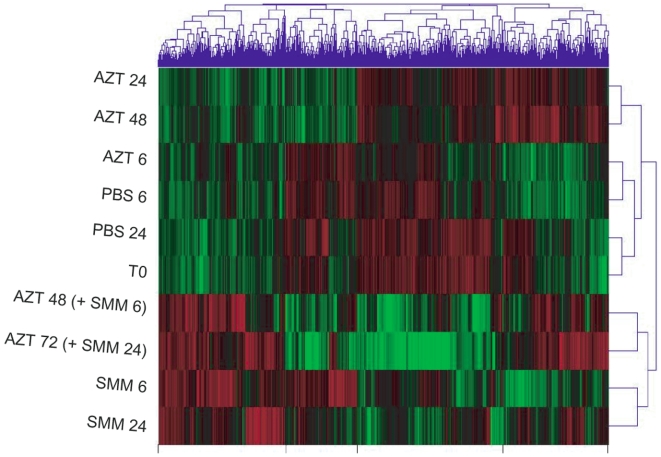
Clustering of the expression data. Clustering of the data, as described in Methods, revealing the general organization of differential gene expression. Green color represents reduced gene expression relative to red color, which represents increased expression. Treatments are explained in Table 1. PBS: phosphate buffered saline. AZT: azithromycin. SMM: Supernatant of mucopurulent material from CF airways.

Clustering of the expression data using the methods described revealed a complex pattern of gene expression changes (Fig. 1). The overall trends that could be derived from the cluster analysis can be summarized as follows:

#### Baseline gene expression

By comparing T0 to the two controls, PBS 6 and PBS 24, it can be appreciated that time of harvest has a measurable effect on global gene expression. While PBS 24 is distinctly similar to T0, PBS 6 is clearly separated. This finding suggests that time after media change during standard culture conditions (6 vs. 24 hr) can affect baseline gene expression values, likely related to alterations in media components over time or diurnal variations. These findings highlight the importance of using controls harvested at the same time during the experiment for the individual treatment comparisons, rather than comparing all treatments to T0, which could lead to false conclusions about treatment effects. All results reported in this study utilize comparisons with appropriate time-matched controls.

#### Effect of AZT alone

AZT alone significantly altered global gene expression at exposures >6 hrs. The close relationship between AZT 6 and PBS 6 in the dendogram indicated very few expression differences between these two treatments. In contrast, AZT treatments for 24 and 48 hrs are clearly distinct from PBS 24 hrs. Most AZT-induced gene expression changes occurred by 24 hours as indicated by the close relationship of AZT 24 to AZT 48, although the degree of induction of some up-regulated genes appears to be greater as exposure time is increased (more intense red in AZT 48 hr as compared to AZT 24 hr).

#### Effect of SMM

SMM treatment alters the global gene expression more extensively than AZT, as indicated by all SMM treatments (regardless of AZT pretreatment) clustering together. This result points to the expected ability of the inflammatory SMM treatment to alter global gene expression.

#### Effect of AZT on SMM

Interestingly, AZT pretreatment, although clearly unable to prevent the strong SMM-induced effect, resulted in noticeable alterations in SMM-induced expression, and close evaluation indicates changes in both directions.

Overall, the ability to detect gene expression differences induced by AZT in both SMM treated and untreated cultures (Table 2), coupled with the complex patterns indicated by the cluster analysis (Fig. 1), led us to seek an understanding of the specific genes/gene families that were altered among relevant treatment/control pairs.

### Gene Ontology (GO) analyses reveal significant trends

**Figure 2:**
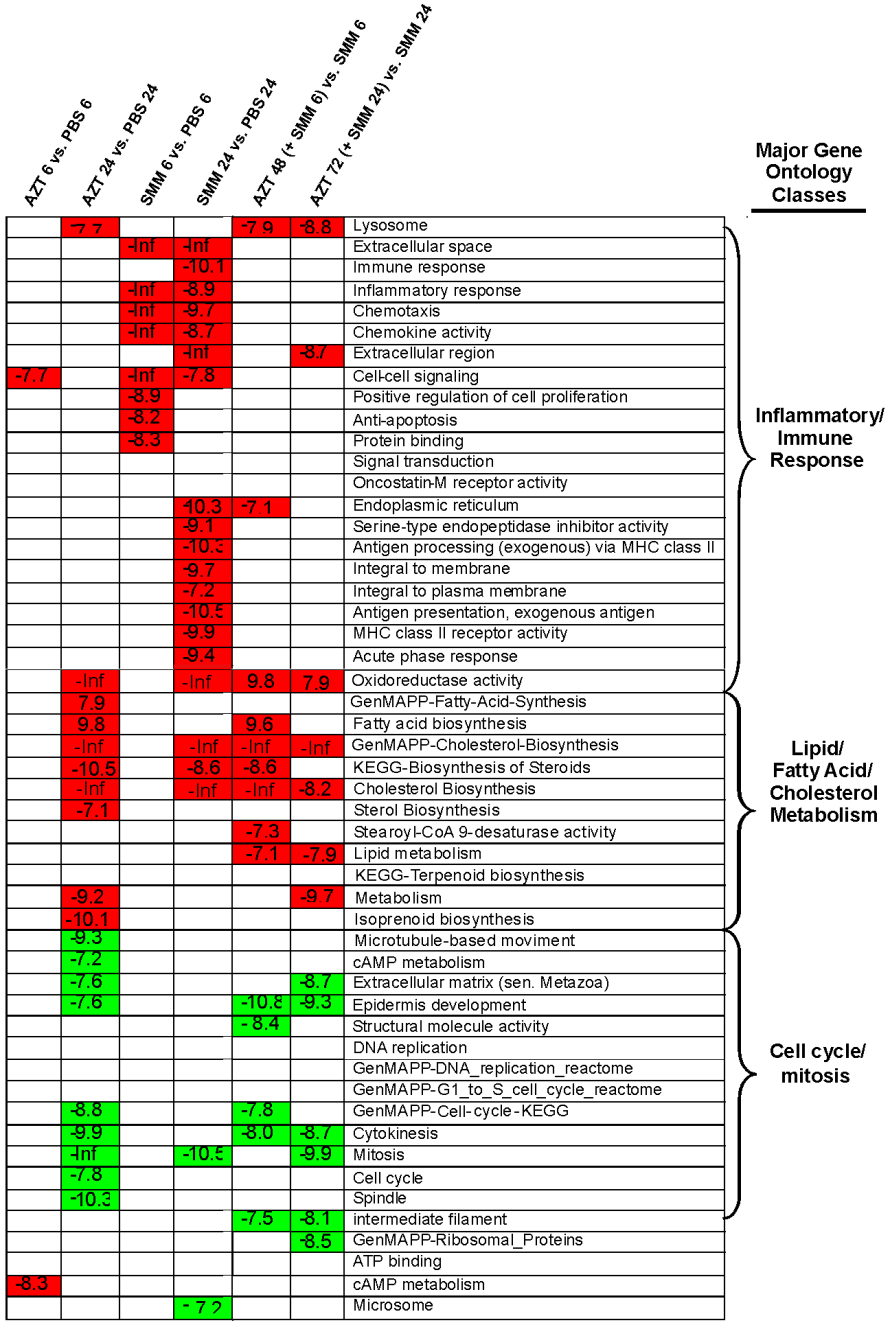
Illustration of the most significant gene ontology (GO) groups whose expression was modulated by the various treatments. The following comparisons were analyzed: AZT 6 hr vs. PBS 6 hr, AZT 24 hr vs. PBS 24 hr, SMM 6 hr vs. PBS 6 hr, SMM 24 hr vs. PBS 24 hr, AZT 48 hr (+SMM during last 6 hr) vs. SMM 6 hr, and AZT 72 hr (+SMM during the last 24 hr) vs. SMM 24 hr. Green = down-regulated groups; red = up-regulated groups; white = not significant. Values in boxes represent the probability that the genes within the list from each GO group would be present by chance – the more negative, the less likely genes within these categories are present by chance. See corresponding text for details.

We identified GO groups whose gene expression patterns were significantly different among treatments. Figure 2 summarizes the findings by indicating all of the most highly significant GO groups for the selected treatment pair comparisons (hypergeometric probability <10^−7^). Both up-regulated (red) and down-regulated (green) GO annotation groups are listed. This summary revealed three major biologically relevant groupings of GO annotations associated with differential expression among treatment pairs (groupings determined subjectively): 1) inflammatory/immune responses, 2) lipid/fatty acid/cholesterol metabolism, and 3) cell cycle/mitosis. Each one of the major groupings will be discussed in brief separately, followed by a more detailed analysis of the findings.

#### Inflammation/immune response genes

As expected, the inflammation/immune response genes were very significantly up-regulated by SMM treatment (Fig. 2). The GO groups can be roughly divided into those that are up-regulated by 6 hrs (e.g., chemotaxis and anti-apoptosis) and those not up-regulated until 24 hr (e.g., antigen processing). AZT alone showed no evidence of significantly affecting these pathways in a global way, with only the cell-cell signaling module showing significant effects, and then only at 6 hrs. Additionally, AZT did not significantly alter the global response of genes in these pathways after exposure to SMM, as indicated by lack of significance in the AZT+SMM vs. SMM columns. Thus, it seemed from the GO analyses as if the cells were able to mount a global inflammatory response to SMM, even in the presence of AZT.

#### Genes involved in lipid/fatty acid/cholesterol metabolism

Various GO classes associated with lipid/fatty acid/cholesterol metabolism were strongly up-regulated by both AZT and SMM after 24 hrs (Fig. 2). Interestingly, despite the fact that the SMM treatment alone could up-regulate genes in these pathways, these GO categories were potentiated by the AZT pretreatment, i.e., they are significantly up-regulated in SMM-exposed HBE pretreated with AZT as compared with SMM alone. This can be clearly appreciated in Fig. 2 as consistent up-regulation of many of these GO groupings in the AZT+SMM vs SMM columns.

#### Genes involved in cell cycle/mitosis

Genes that fell within GO groups related to DNA replication, cell cycle, and mitosis tended to be significantly down-regulated by AZT treatment. As with the lipid/fatty acid/cholesterol GO categories, this effect of AZT alone was also seen in cells treated with SMM, as indicated by significant down-regulation of these GO groups in the AZT+SMM vs SMM columns (Fig. 2).

### Detailed analyses of specific genes within significant GO categories

#### Inflammation/immune response genes

**Figure 3:**
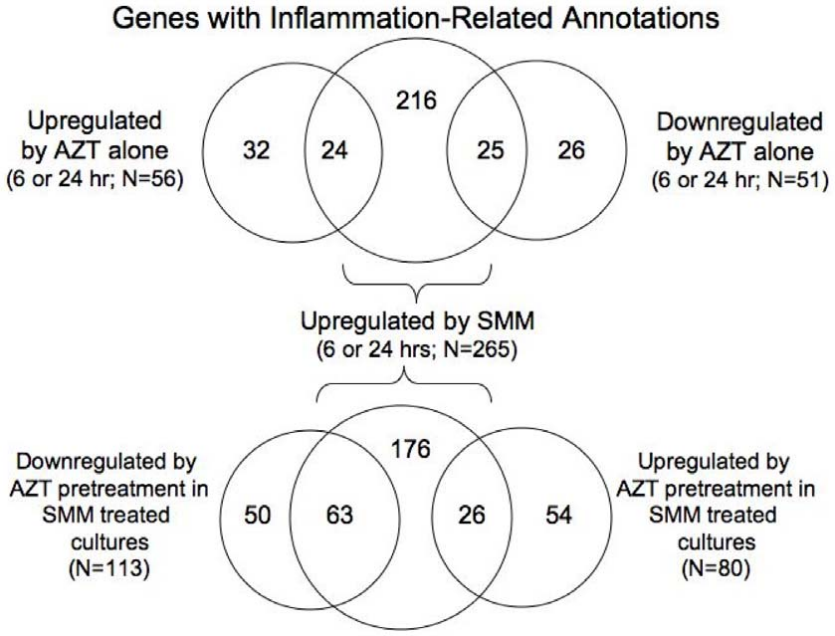
Venn diagrams representing the number of differentially expressed inflammation-related genes as a function of treatment. The top Venn diagram compares SMM-induced genes to AZT up- and down-regulated genes. The bottom Venn diagram shows how AZT pretreatment affected SMM genes.

As shown in Fig. 2, there was little indication from the global analysis of mRNA expression data that AZT pretreatment led to across-the-board alterations in gene expression of classic immune/inflammatory response genes. However, our interest in the inflammatory response of AZT and the extensive literature linking AZT to inflammatory processes in the lung prompted a closer evaluation of the effect of AZT treatment on specific mRNAs with predicted inflammatory/immune functions. To accomplish this, we looked specifically at the differentially regulated genes in our study (pulled from the probesets described in Table 2 and given in Supporting Information, Table S2) that were annotated in various GO categories known to be affected by inflammatory stimuli (see caption to Table 3). The results of these efforts are shown in Tables 3–5, Fig. 3, and Online Supporting Information, Table S3.

**Table 3:** Differential regulation of genes annotated in inflammatory/immune/defense*-related pathways as a function of treatment group.

Treatment Comparison	Total number of DR* genes	Number of DR IID* genes		Percent of all DR genes identified as IID*
AZT 6 hr vs PBS 6 hr	58	25	**Total**	**53%**
			Up-regulated	33%
			Down-regulated	20%
AZT 24 hr vs PBS 24 hr	116	85	**Total**	**73%**
			Up-regulated	34%
			Down-regulated	39%
SMM 6 hr vs PBS 6 hr	345	211	**Total**	**61%**
			Up-regulated	45%
			Down-regulated	16%
SMM 24 hr vs PBS 24 hr	297	193	**Total**	**65%**
			Up-regulated	46%
			Down-regulated	19%
AZT 48 hr (+SMM 6 hr) vs SMM 6 hr	205	135	**Total**	**66%**
			Up-regulated	31%
			Down-regulated	35%
AZT 72 hr (+SMM 24 hr) vs SMM 24 hr	143	110	**Total**	**77%**
			Up-regulated	45%
			Down-regulated	32%

* IID = inflammatory/immune/defense related genes. DR = differentially-regulated. Genes were entered into this table as inflammation-related if the z.pv was significant (<0.05) and if the Gene Ontology classifications contained one or more of the following: cell adhesion, proteolysis, MAPK or MAPKKK, inflammatory/defense/immune/acute-phase response, apoptosis/cell death, protein transport, cytokine activity, nitric oxide, NF-kappaB, antimicrobial humoral response, unfolded protein, phagocytosis, B cell activation, lymphocyte proliferation, protein transport/secretion/folding. Note: This table reflects genes, not probesets; thus, the numbers differ from Table 2. Some of the significant genes are represented by multiple significant probesets. Genes not annotated as inflammation-related are either unannotated or had Gene Ontology classifications that do not contain the annotations listed above. This table does not include differentially expressed genes in the cholesterol/fatty acid metabolic pathways or the cell cycle/mitosis/cell division pathways that are discussed in detail elsewhere in the manuscript. The table represents data from a total of 395 inflammation-related genes that were found to be differentially-regulated based upon our established criteria.

In contrast to the global analyses described above, careful examination of specific, selected annotation groups indicated that the genes differentially regulated by AZT did, in fact, tend to encompass those with inflammatory-related annotations. This is reflected by the high percentage of all significantly up- or down-regulated genes that carry inflammatory-related annotations, with values ranging from 53–77% (Table 3) – percentages much higher than would be expected by chance for the relatively small number of selected annotation groups (total number of annotation groups = 20). Enrichment of inflammation-related annotations was expected with SMM-treated cultures, since SMM is a potent inflammatory stimulus, but it was also seen with AZT treatment, both in the presence and absence of SMM. Thus, genes regulated by both SMM and AZT fall to a significant degree into inflammatory-type pathways, despite the lack of significance when analyzed by GO classification.

As predicted, SMM at both 6 and 24 hrs was a stronger inflammatory stimulus than AZT, as indicated by the larger number of inflammatory-related genes that were altered in expression after the SMM treatment as compared with the AZT treatment (Table 3). As expected, most of these inflammatory-related genes after SMM treatment were up-regulated (94.4 and 88% for 6 and 24 hr, respectively; Table 4). In contrast, only 46% of the inflammatory-related genes affected by AZT were up-regulated after 24 hrs, with the rest being down-regulated. This same trend for AZT toward down-regulation of inflammatory genes was evident when AZT treatment preceded the exposure of HBE to SMM. In these comparisons, 58% of inflammation-related genes that were differentially expressed between SMM-treated cultures and SMM-treated cultures pretreated with AZT were in the down-regulated category (Table 4; last column).

**Table 4 pone-0005806-t004:** Inflammation-related genes classified as up- or down-regulated as a function of treatment comparison.

Treatment Comparison	Category	Number	Percent
AZT 6 hr vs PBS 6 hr	Up-regulated	19	76%
	Down-regulated	6	24%
AZT 24 hr vs PBS 24 hr	Up-regulated	40	46%
	Down-regulated	45	54%
SMM 6 hr vs PBS 6 hr	Up-regulated	199	94%
	Down-regulated	12	6%
SMM 24 hr vs PBS 24 hr	Up-regulated	170	88%
	Down-regulated	23	12%
AZT 48 hr (+SMM 6 hr) vs SMM 6 hr	Up-regulated	56	42%
	Down-regulated	79	58%
AZT 72 hr (+SMM 24 hr) vs SMM 24 hr	Up-regulated	46	42%
	Down-regulated	64	58%

* See Table 3 for information concerning the selection of these genes as inflammation-related. “Percent” in this table represents the percentage of these inflammation-related genes that are either up- or down-regulated (category).

We next used Venn diagrams, which help to visualize the commonality of differential expression between different treatment pairs, to comprehend whether or not the inflammatory genes induced by AZT were the same genes as those induced by SMM. We found that only 24 (9%; n = 24) of genes up-regulated by 6 or 24 hr SMM treatment were also up-regulated by AZT treatment (Fig. 3, upper Venn diagram, left). These same 24 genes represented 43% of all AZT-induced up-regulated genes. At first glance, this might suggest a tendency for AZT treatment to stimulate production of inflammatory-related genes that are also induced by SMM, but with SMM being a much stronger stimulus. However, a similar number of SMM-induced inflammatory-related genes (9%) was also down-regulated by AZT treatment (Fig. 3, upper Venn diagram, right).

AZT pretreatment also affected SMM-induced inflammatory genes (Fig. 3, bottom Venn diagram). Nearly 24% (n = 63) of all genes normally up-regulated by SMM at either 6 or 24 hrs were significantly attenuated in expression by either one or both of the AZT pretreatments (left), suggesting that AZT can dampen the ability of SMM to induce these genes. Only 9.8% (n = 26) of the same genes were increased in expression by AZT pretreatment (right). While the qualitative and quantitative characteristics of these up- and down-regulated genes are likely to be important, as discussed below, the overall trend was towards a more anti-inflammatory effect of AZT.

Because AZT could affect genes annotated in inflammatory categories in either direction, it became important to look specifically at the genes involved. Table 5 shows the differential regulation for a selected series of these genes (all inflammatory-related genes are shown in Supporting Information, Table S3). As expected, cytokines and chemokines were up-regulated by SMM, but the effect of AZT on these genes was variable and inconclusive. IL-8, which has been previously shown to be either up- or down-regulated by AZT [30]–[33] was significantly increased only after short term exposure to AZT alone, and AZT did not potentiate gene expression of IL-8 in the presence of SMM exposure (Table 5).

**Table 5:** Response of cytokine/chemokine genes and their receptors for the selected treatment pairs.

Gene Name	Gene Symbol	AZT 6 vs PBS6		AZT24 vs PBS24	SMM6 vs PBS6	SMM24 vs PBS24			AZT48 SMM6 vs SMM6	AZT72 SMM24 vs SMM24
chemokine (C-X-C motif) ligand 1	CXCL1				UP		UP			
chemokine (C-X-C motif) ligand 14	CXCL14									DOWN
chemokine (C-X-C motif) ligand 16	CXCL16						UP			
chemokine (C-X-C motif) ligand 2	CXCL2				UP		UP			
chemokine (C-X-C motif) ligand 3	CXCL3		DOWN		UP		UP			DOWN
chemokine (C-X-C motif) ligand 5	CXCL5				UP		UP			
chemokine (C-X-C motif) ligand 6	CXCL6		DOWN		UP		UP	DOWN		
chemokine (C-X-C motif) receptor 7	CXCR7	UP			UP					
interleukin 13 receptor, alpha 1	IL13RA1							UP		
interleukin 17C	IL17C				UP					
interleukin 19	IL19				UP					
interleukin 1, alpha	IL1A				UP		UP	DOWN		UP
interleukin 1, beta	IL1B				UP					
interleukin 1 family, member 9	IL1F9				UP		UP			
interleukin 1 receptor-like 1	IL1RL1							DOWN		
interleukin 1 receptor antagonist	IL1RN	UP	UP		UP		UP			
interleukin 32	IL32				UP		UP			
interleukin 4 receptor	IL4R				UP		UP			
interleukin 6 (interferon, beta 2)	IL6				UP		UP	UP		
interleukin 6 signal transducer	IL6ST	UP			UP		UP	UP		
interleukin 7	IL7		DOWN				DOWN			
interleukin 8	IL8	UP			UP		UP			

The direction of differential regulation is given by UP or DOWN, indicating the direction that gene expression moved in the treatment (listed first) vs. the control (listed last). Genes are only considered differentially-regulated if the LPE z.pv<0.05 as determined as described in Methods. Only genes with significant findings for at least one of the comparisons are shown (genes in the same categories that were not affected by any of the treatments are not shown). This Table is a subset of Table S3 from Supporting Information, which lists all genes with inflammatory annotations. Blank cells indicate that the gene was not-differentially-regulated for that particular treatment comparison.

In contrast to cytokines, some genes stimulated by SMM were attenuated by exposure to AZT (Table 6; Supporting Information, Table S3). Of particular interest, both MUC5AC and MMP9 expression levels were significantly reduced by AZT, confirming previous findings with other macrolides [19]–[22]. In fact, for these two genes, pretreatment with AZT completely prevented the normally expected up-regulation by SMM and estimated gene expression values were reduced to baseline values or below (not shown). Interestingly, while MUC5AC was the only mucin gene significantly altered by AZT exposure, several MMPs (MMP1, 2, 10, 13, and 28) in addition to MMP9 tended to be down-regulated by AZT. The same general pattern of attenuation by AZT was noticed for SERPIN genes.

**Table 6:** Response of selected classes of inflammation-related genes across selected treatment pairs.

Gene Class and Response/Gene Name	Gene Symbol	AZT 6 vs PBS6	AZT24 vs PBS24	SMM6 vs PBS6	SMM24 vs PBS24	AZT48 SMM6 vs SMM6	AZT72 SMM24vs SMM24
MHC Class II complex genes: Up-regulated by SMM: potentiated by AZT							
major histocompatibility complex, class II, DP beta 1	HLA-DPB1				UP		
major histocompatibility complex, class II, DQ alpha 1	HLA-DQA1		DOWN		UP		UP
major histocompatibility complex, class II, DQ beta 1	HLA-DQB1			UP	UP	UP	UP
major histocompatibility complex, class II, DR alpha	HLA-DRA				UP		UP
major histocompatibility complex, class II, DR beta 1	HLA-DRB1				UP	UP	UP
Matrix metalloproteinases: tend to be up-regulated by SMM treatment, down-regulated by AZT							
matrix metallopeptidase 1	MMP1		DOWN		UP	DOWN	
matrix metallopeptidase 2	MMP2						DOWN
matrix metallopeptidase 9	MMP9		DOWN	UP		DOWN	DOWN
matrix metallopeptidase 10	MMP10		DOWN	UP	UP	DOWN	DOWN
matrix metallopeptidase 13	MMP13		DOWN	UP	UP	DOWN	DOWN
matrix metallopeptidase 14	MMP14				UP		
matrix metallopeptidase 28	MMP28				UP		DOWN
SERPIN genes: tend to be upregulated by SMM; attenuated by AZT							
serpin peptidase inhibitor, clade A, member 1	SERPINA1				UP		
serpin peptidase inhibitor, clade A, member 3	SERPINA3			UP	UP		UP
serpin peptidase inhibitor, clade B, member 1	SERPINB1			UP	UP	DOWN	DOWN
serpin peptidase inhibitor, clade B, member 2	SERPINB2		DOWN			DOWN	DOWN
serpin peptidase inhibitor, clade B, member 4	SERPINB4			UP	UP		
serpin peptidase inhibitor, clade B, member 9	SERPINB9			UP	UP		DOWN
serpin peptidase inhibitor, clade E, member 1	SERPINE1			UP	UP	DOWN	
Mucins: Response variable: Up-regulated by SMM; strong inhibition of MUC5AC by AZT							
mucin 5AC	MUC5AC			UP	UP	DOWN	DOWN
mucin 5B	MUC5B				UP		
mucin 1	MUC1				UP		
mucin 4	MUC4				UP		
mucin 13	MUC13				UP		
Miscellaneous genes; Generally upregulated by SMM; Mostly potentiated or by AZT							
dual specificity phosphatase 5	DUSP5	UP	UP	UP	UP		
lymphocyte antigen 96	LY96		UP	UP	UP	UP	UP
transferrin receptor (p90, CD71)	TFRC		UP		UP	UP	UP
ferritin, heavy polypeptide 1	FTH1		UP		UP		
ferritin, light polypeptide	FTL		UP			UP	UP
growth differentiation factor 15	GDF15		UP	UP	UP	UP	UP
hypoxia-inducible factor 1, alpha subunit	HIF1A					UP	UP
vascular endothelial growth factor A	VEGFA			UP	UP		UP
plasminogen activator, tissue	PLAT		DOWN	UP	UP	DOWN	DOWN
plasminogen activator, urokinase	PLAU			UP	UP		UP

The direction of differential regulation is given by UP or DOWN, indicating the direction that gene expression moved in the treatment (listed first) vs. the control (listed last). Genes are only considered differentially-regulated if the LPE z.pv <0.05 as determined as described in Methods. Only genes with significant findings for at least one of the comparisons are shown (genes in the same categories that were not affected by any of the treatments are not shown). This Table is a subset of Table S3 from Supporting Information, which lists all genes with inflammatory annotations. Blank cells indicate that the gene was not-differentially-regulated for that particular treatment comparison.

Other inflammation-related genes were specifically up-regulated by AZT. These genes included MHC Class II genes, genes involved in iron metabolism (ferritin, transferrin receptor), hypoxia responses (HIF1A, VEGFA), and several others (Table 5). Plasminogen activator (PLAT) was an additional gene of interest whose expression was attenuated by AZT, but its sister gene, PLAU, was potentiated by AZT.

### Lipid/Fatty Acid/Cholesterol Metabolism Genes

**Figure 4:**
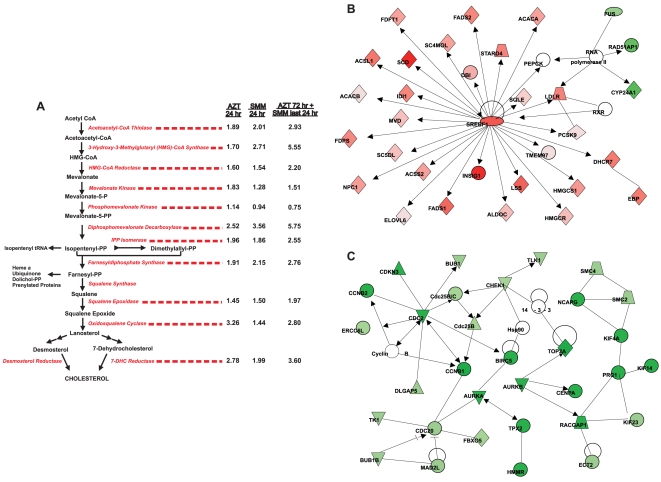
AZT up-regulates lipid/cholesterol metabolism and down-regulates cell cycle genes. A: The cholesterol biosynthetic pathway and the fold-changes induced for each of the enzymes within the pathway are indicated. Data are expressed as fold changes in gene expression values from 24 hr AZT vs. 24 hr PBS (left numbers), 24 hr SMM vs. 24 hr PBS (center numbers) and 72 hr AZT (+addition of mucosal SMM during the last 24 hr) vs. 24 hr PBS (right numbers). Both SMM and AZT increased the enzymes involved in the cholesterol biosynthetic pathway, and their effect appears to be additive. B and C: Ingenuity Pathways Analysis™ was used to generate two top significant networks for genes regulated by AZT 24 hr vs. PBS 24 hr. The top two networks are shown. The analysis settings were as follows: only direct relationships were considered, endogenous chemicals were excluded, and only relationships where data sources = Argonaute 2 or Ingenuity curated findings were considered. The network depicted in B was labeled “Lipid Metabolism, Small Molecule Biochemistry, Nucleic Acid Metabolism” and had a score = 47. The network depicted in C was labeled “Cancer, Cell Cycle, Reproductive System Disease” and had a score = 44. Red indicates up-regulation. Green indicates down-regulation.

The striking up-regulation of these pathways by AZT after 24 hrs represents a novel finding. A detailed view of genes in these GO categories that were significantly affected by the treatments in this study is provided in the Supporting Information, Table S4. It can be appreciated that both lipid/fatty acid/cholesterol metabolic/biosynthetic genes and genes related to lipid/cholesterol transport are affected by AZT. A specific analysis of a subset of these genes, the cholesterol biosynthetic pathway, as viewed schematically in Figure 4A, provides further confirmation of the findings. Many of the genes up-regulated by AZT were also up-regulated by SMM, and the results suggest an additive effect, since many of the genes were highest in expression in the presence of both SMM and AZT (Supporting Information, Table S4).

Of particular interest in this group was the up-regulation of SREBP1 (sterol regulatory element binding protein 1. SREBP1, and its counterpart SREBP2, are structurally related proteins that control cholesterol homeostasis by stimulating transcription of sterol-regulated genes containing the sterol regulatory element. Up-regulation of this transcription factor alone could, at least in part, explain the up-regulation of many of the other genes in this pathway, a finding that was illustrated in a schematic view of the most significant pathways altered by 24 hr AZT treatment as determined by Ingenuity Pathway Analysis™ software (Figure 4B). Additionally, a second master regulator of cholesterol homeostasis, insulin-induced gene-1 (INSIG-1), which is a critical component of the sterol-sensing pathway due to its modulation of SREBP processing that prevents SREBP1 activation [34], was also significantly up-regulated by the AZT treatment (Supporting Information, Table S4), suggesting that the cells were attempting to reestablish equilibrium.

### Cell Cycle/Mitosis Genes

The gene expression values of the cell cycle/mitosis genes, including genes for DNA replication and chromosome segregation, were down-regulated by AZT treatment, mainly when AZT was present for more than 6 hours (Supporting Information, Table S5). Interestingly, upon more careful evaluation, many of these same genes (43%) having the cell cycle/mitosis annotations that were down-regulated by AZT at 24 hr were also significantly down-regulated by 24 hr SMM treatment, although the significance of the down-regulation after the SMM treatment was not as high as with the AZT treatment, as can be appreciated by lack of significant findings for SMM treatments in Fig. 1. The effect of AZT after 24 hrs on these genes could be visualized schematically utilizing the Ingenuity Pathways Analysis™ output (Figure 4B). For the second most highly significant network, nearly all of the genes represented as green (down-regulated) were found in the GO categories highlighted in Supporting Information, Table S5.

### Confirmatory Studies

To confirm and/or extend some of the major findings in the microarray studies, we have analyzed three specific components: MUC5AC expression, IL-8 secretion, and cholesterol metabolism. A brief summary of these results follows.

#### AZT inhibits basal and SMM-stimulated mucin production

**Figure 5:**
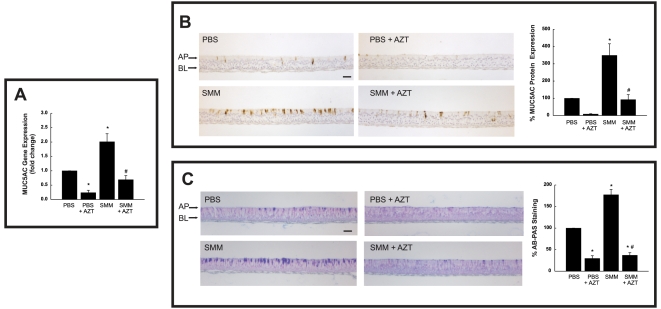
AZT decreases basal and SMM-stimulated mRNA and protein expression levels of MUC5AC in human airway epithelia. Well-differentiated HBE were exposed for 24 hr to mucosal PBS or mucosal SMM in the absence of presence of 72 hr pretreatment with 30 µg/ml serosal AZT. A: MUC5AC mRNA levels, expressed as fold change from mucosal PBS-exposed HBE. B: Immunocytochemical assessment of MUC5AC in HBE. Right panel depicts compiled data from MUC5AC expression as a percent of PBS-exposed HBE. C: AB-PAS staining from HBE subjected to the various treatments. Right panel shows compiled data from AB-PAS staining as a percent of PBS-exposed HBE. *p<0.05 vs. PBS-exposed HBE; # p<0.05 SMM+AZT-exposed HBE vs. SMM-exposed HBE.

The inhibitory effect of AZT on MUC5AC gene expression seen on the microarrays was reproduced by quantitative real-time PCR studies (Fig. 5A). To confirm that the inhibitory effect of AZT was coupled to a decreased MUC5AC protein expression, immunocytochemical analysis for MUC5AC was performed. It was found that AZT decreased MUC5AC protein expression levels from both PBS- and SMM-exposed cultures (Fig. 5B). A similar effect of AZT on total mucin protein expression was observed in HBE subjected to the same experimental protocol and stained with Alcian blue/periodic acid-Schiff (AB/PAS; Fig. 5C). AB/PAS stains carbohydrates and reveals mucin-secreting cells, since ∼90% of the mucin weight is derived from glycans. Thus, in the case of MUC5AC, the microarray data were corroborated by protein expression analyses.

#### Azithromycin pretreatment increases IL-8 release after inflammatory challenge

**Figure 6:**
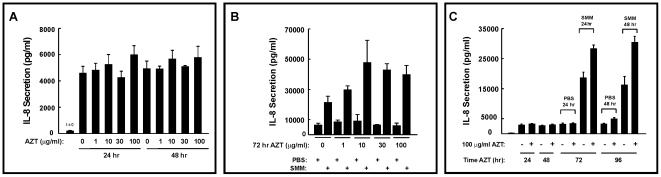
Effect of AZT on IL-8 secretion in human airway epithelia. A: AZT does not affect basal airway epithelial IL-8 secretion. Basal IL-8 secretion from well-differentiated primary cultures of normal human bronchial epithelia serosally exposed to different doses of AZT for 24 hr and 48 hr. B: Dose response for AZT-potentiated IL-8 secretion triggered by 24 hr mucosal exposure of HBE to supernatant from mucopurulent material (SMM) from CF airways. Well-differentiated primary cultures of normal human bronchial epithelia were pretreated with AZT for 72 hr and exposed to mucosal PBS or SMM during the last 24 hr of macrolide treatment. C: Time course for AZT-potentiated SMM-induced IL-8 secretion. Vehicle or 100 µg/ml AZT were added to the serosal surface of well-differentiated normal HBE and a 48 hr time course performed for IL-8 secretion. AZT pretreatment did not alter the basal levels of secreted IL-8, but potentiated SMM-induced IL-8 secretion at both 24 and 48 hr (corresponding to 72 and 96 hr AZT treatment, respectively).

As determined by microarrays, IL-8 levels were not dramatically affected and these results were confirmed by real-time PCR analysis (data not shown). However, because of the reported alterations in IL-8 levels after AZT treatment [31], we evaluated whether IL-8 secretion correlated with the mRNA findings. To begin these studies, IL-8 protein levels were measured in the presence or absence of AZT as described in methods. Figure 6A illustrates that none of the AZT doses had any significant effect on the basal IL-8 secretion, regardless of the duration of exposure, consistent with the microarray results. Surprisingly, however, additional studies using SMM to stimulate IL-8 levels showed that AZT enhanced SMM-induced IL-8 secretion, an effect that was apparent at 1 µg/ml AZT and reached a maximum at 10 µg/ml AZT (Fig. 6B).

To further characterize this response, longer time courses were performed in HBE exposed to a maximal dose of 100 µg/ml AZT for up to 96 hrs, and IL-8 secretory responses in the absence or presence of SMM were evaluated. AZT again had no effect on basal levels of secreted IL-8 at 24 or 48 hr (Fig. 6C). IL-8 secretion triggered by 24 or 48 hr SMM exposure was potentiated by pretreatment with AZT (Fig. 6C). Thus, in contrast to the microarray data (Table 5), which showed no effect of AZT pretreatment on IL-8 at the mRNA level, AZT pretreatment, surprisingly, altered IL-8 protein levels. These findings highlight additional complexities involved in evaluating the inflammatory effects of macrolides and in using microarray data to evaluate an inflammatory state.

#### Cholesterol metabolic pathway

**Figure 7:**
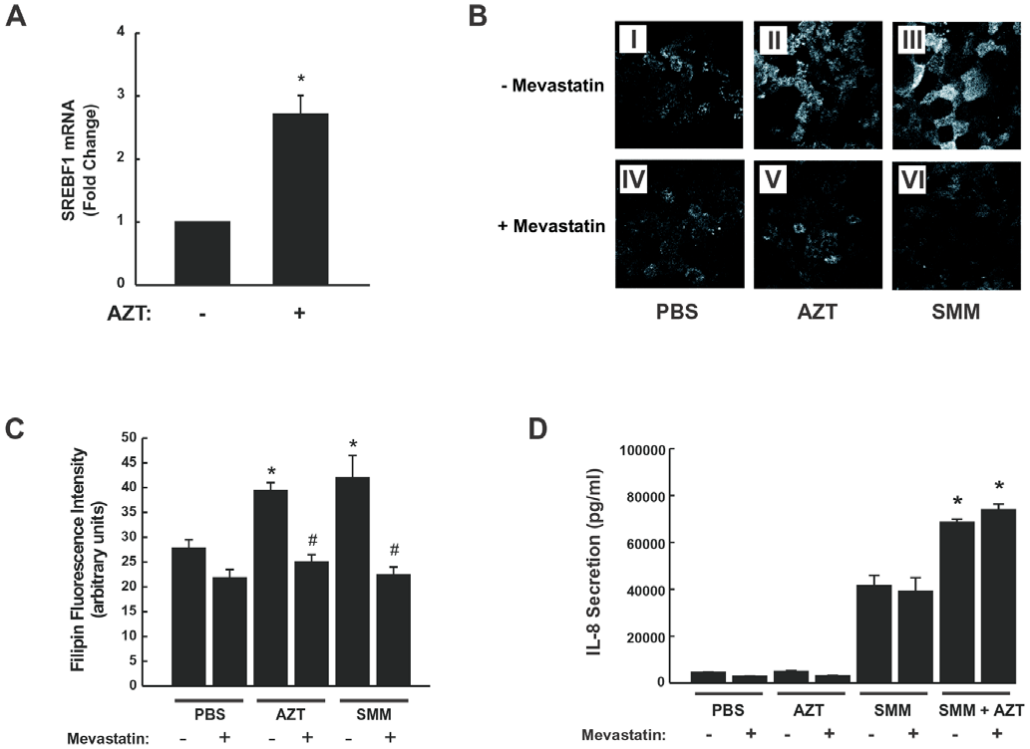
Mucosal bacterial and inflammatory mediator challenge (SMM) or serosal AZT induces accumulation of cholesterol in human airway epithelia. A: Quantitative RT-PCR confirmation of increased SREBP1 levels in AZT-treated cultures. B: Representative filipin stain in WD HBE exposed to 24 hr mucosal PBS (I), 24 hr mucosal SMM (II) or 24 hr 30 µg/ml serosal AZT (III). (IV–VI): Same conditions as in I–III, except that cultures were pretreated with 50 µM mevastatin, as described in Methods. Bar: 10 µm. C: Compiled data from filipin fluorescence from the treatments illustrated in 7B. *p<0.05 vs. 24 hr PBS-exposed HBE; # p<0.05 vs. same condition without mevastatin pretreatment. D: IL-8 secretion induced by SMM or by SMM in presence of AZT is not affected by inhibition of cholesterol biosynthesis. Well-differentiated HBE were exposed for 24 hr to mucosal PBS, mucosal SMM or 30 µg/ml serosal AZT in the presence or absence of mevastatin, and IL-8 secretory responses measured as described in Methods. *p<0.05 SMM+AZT- vs. SMM-exposed HBE.

The microarray results illustrating the up-regulating effect of AZT on the cholesterol metabolic pathway (Fig. 4A) were confirmed using RT-PCR for SREBP1 on a separated set of HBE cultures. These studies demonstrated that the master regulator of cholesterol biosynthesis was up-regulated by AZT (Fig. 7A). To address whether the up-regulation of SREBP1 and cholesterol biosynthetic genes by either AZT or SMM resulted in an increased cholesterol accumulation, HBE were exposed for 24 hr to 1) mucosal PBS, 2) 30 µg/ml serosal AZT, 3) mucosal SMM, or 72 hr 30 µg/ml serosal AZT (with mucosal SMM added during the last 24 hr), followed by staining with filipin to visualize free cholesterol. As depicted in Fig. 7B, filipin fluorescence was equally increased by AZT or SMM exposure in HBE (Figs. 7B-II and 7B-III), as compared with PBS-exposed epithelia (Fig. 7B-I). To confirm that AZT- or SMM-increased filipin fluorescence resulted from cholesterol biosynthesis, cultures were pretreated with 50 µM mevastatin to inhibit the activity of 3-hydroxy-3-methylglutaryl-CoA reductase. Mevastatin pretreatment blocked the effect of AZT or SMM on filipin fluorescence (Figs. 7B-V and 7B-VI), confirming that exposure of HBE to AZT or SMM increases cholesterol biosynthesis. The compiled data from filipin fluorescence intensity from all conditions are depicted in Fig. 7C. Exposure of HBE to both AZT and SMM did not further increase filipin fluorescence as compared to filipin fluorescence from HBE exposed to AZT or SMM alone (data not shown).

We then considered whether the increase in cholesterol biosynthesis was linked to the increased IL-8 secretion shown in Fig. 6. To address this question, IL-8 secretory responses were investigated in HBE individually exposed to PBS, AZT or SMM or HBE exposed to both AZT+SMM in the absence or presence of mevastatin. Although mevastatin decreased AZT- or SMM-induced cholesterol accumulation in HBE (Figs. 7B and 7C), it did not affect SMM-induced IL-8 secretion or decreased AZT-potentiated SMM-dependent IL-8 secretion (Fig. 7D). These findings suggest that there is no link between cholesterol accumulation resulting from AZT or SMM and IL-8 secretion. These results also suggest that the potentiating effect of AZT on SMM-induced IL-8 secretory responses (Fig. 6) is not mediated by increases in cholesterol biosynthesis.

## Discussion

The reported effects of macrolides on inflammatory responses have recently been reviewed [3]. They include suppression of production and secretion of pro-inflammatory cytokines, suppression of inducible nitric oxide (NO) synthase-mediated NO production, decreased mucus synthesis and secretion, promotion of inflammatory cell apoptosis, decrease in production of nuclear transcription factors, inhibition of chloride and water secretion across airways, and interruption of bacterial virulence [3]. In our studies, the combined data, primarily obtained from RNA microarrays using a relevant model of well-differentiated primary airway epithelial culture, demonstrate that the macrolide antibiotic AZT affected the expression of genes predominantly in three major categories: inflammation/immune response (variable response, both up and down-regulation), lipid/fatty acid/cholesterol metabolism (up-regulation), and cell cycle/mitosis (down-regulation). Whereas the effect on inflammation/immune responses and cell cycle/mitosis genes may have been predicted from previous work, the strong effect on lipid metabolism genes is a novel finding.

Our analyses of expression for genes annotated in inflammatory/immune pathways after AZT treatment point to complex and gene-specific effects. Unlike the inflammatory stimulus SMM, which primarily increased the expression of genes annotated in inflammatory-related pathways, AZT differentially regulated inflammatory-related genes in both directions (up and down), with a trend toward down-regulation based upon numbers (Fig. 3 and Tables 3 and 4). This finding is significant because it suggests that the induction of at least some inflammatory genes normally strongly up-regulated by SMM was reduced by the AZT pretreatment. It also helps explain why the AZT effect was not evident in the global analyses, because a dilution of GO annotations into both up- and down-regulated categories would tend to lower the significance of each effect individually. AZT did not have a consistent effect on cytokine/chemokine expression (Table 5), which suggests that classic mechanisms leading to up-regulation of cytokines/chemokines in airway epithelial cells, such as activation of the NF-kappaB-mediated pathways, are, for the most part, unaffected by AZT. Nevertheless, AZT had significant effects on other highly relevant genes known to be involved in airway biology during the course of inflammatory disease. Overall, the microarray data provides a very complex picture of the effect of AZT on inflammatory-related genes, and it is likely that the individual gene effects, such as down-regulation of MUC5AC and MMP9, may be more important to the final inflammatory outcome than the overall global response might predict. The results are consistent with the literature findings suggesting that AZT can display anti-inflammatory properties at the same time that some inflammatory processes seem to be up-regulated.

Our microarray studies (Table 6) have confirmed and extended previous findings showing decreased gene expression of MUC5AC and MMP9 after macrolide treatment. In agreement with our observations, inhibitory effects of macrolides on inflammatory mediators were observed in the presence of inflammatory stimuli, such as lipopolysaccharide [19]–[22], [35], [36]. The inhibitory effect of AZT on MUC5AC gene expression in the present study was confirmed at the protein level (Fig. 5). This finding may explain the link between macrolide use and decreased mucus secretion in a variety of chronic airway diseases [37].

In addition to MMP9, our studies revealed that other MMP proteins (e.g., MMP1, 10, and 13) were also inhibited by AZT (Table 5). MMP9, which is involved in the degradation of extracellular matrix components, was reported to mediate N-terminal cleavage of IL-8, to potentiate IL-8 activation of neutrophils [38], and to cleave and activate latent TGF-β2 [39]. Increased concentrations of MMP9 in asthmatic and CF patients as compared to normal subjects [40], [41] and in CF patients with poor lung function [42] have led to the conjecture that MMP9 may contribute to both inflammation and airway remodeling. Because SERPINB (serine peptidase inhibitor Clade B) genes, which are also recognized as important mediators of inflammatory responses in the airway [43], tended to be down-regulated by AZT, our findings suggest that a common down-regulatory mechanism for AZT might be shared with MMP and SERPIN genes (see also Supporting Information, Table S3). The other observed, specific effects of AZT on expression of other inflammatory genes, such as up-regulation of HLA, LY96, GDF15, HIF1A, ferritin and plaminogen activator genes (Table 6), may also be relevant to airway inflammatory diseases. Although we did not directly measure MMP9 protein levels or activity in this study, reduction in MMP9 activity has been measured in recent reports evaluating macrolide activity in *in vivo* systems [44]–[46]. Specifically, of interest with regards to lung disease are the findings that MMP9 concentrations were reduced in sputum from a group of patients with refractory asthma that were receiving clarithromycin [45].

On the other hand, an important aspect of our studies was the demonstration that the inflammatory state could not be predicted from gene expression data alone. This notion was based on the studies conducted using a different measure of airway epithelial inflammation, secreted IL-8 protein levels (Fig. 6). Whereas AZT alone produced no striking effect on secreted IL-8 levels, AZT pretreatment resulted in an unexpected, but consistent, increase in the levels of secreted IL-8 after SMM treatment (Figs. 6B and 6C). This finding is in general agreement with Shinkai *et al* [31], who reported that treatment with AZT for 24–48 hrs increased basal IL-8 release and had an additive effect on LPS-induced IL-8 secretion in normal human bronchial epithelia. In addition, this observation correlates with short-term clinical studies that suggest a pro-inflammatory response to AZT [32]. The increased IL-8 protein secretion was not reflected in the mRNA levels, as determined by microarrays or quantitative PCR. Dissecting out the mechanism responsible for the disconnect between mRNA and protein, which likely involves regulation of post-translational processes and/or IL-8 secretion, will be an important step in understanding the mechanisms of action of the immunomodulatory effects of macrolide therapy.

Unlike genes annotated to inflammation/immune response pathways, the direction of differential gene expression for cholesterol/lipid metabolism and cell cycle genes was very clear (Figs. 2 and 4; Supporting Information, Tables S4 and S5). Lipid/cholesterol related genes were up-regulated by AZT, which constitutes a novel finding in airway epithelial cells, whereas cell cycle genes were down-regulated by the macrolide. Although it is not clear how these changes could contribute to long-term *in vivo* responses, they likely play an important role in the therapeutic mode of action of macrolides.

Perturbations in lipid/cholesterol metabolism have been associated with many diseases (reviewed in [47], [48]), and it is certainly clear that these pathways, and the products produced from them, can regulate inflammation in both a positive and a negative direction. The link between macrolide treatment and lipid/cholesterol pathway gene expression seen in the present study is in agreement with previous observations demonstrating increased total cell phospholipids and cholesterol in rat fibroblasts following macrolide administration [49]. Based on our findings that AZT increases the expression of SREBP1, an important transcriptional regulator of lipid/cholesterol homeostasis, we speculate that the up-regulation of SREBP1 gene expression likely mediates the increased cholesterol biosynthetic response induced by AZT (Figs. 4 and 7; Supporting Information, Table S4). In general, cholesterol levels are a critical determinant of SREBP activation, which involves cleavage of an endoplasmic reticulum-bound precursor into a nucleus-localized transcription factor during periods of cholesterol depletion. Once cholesterol levels normalize, SREBP binds to INSIG, another gene also found to be up-regulated in this study by AZT, to prevent further activation. The up-regulation of the SREBP inhibitor INSIG in our study likely reflects the epithelial need to reestablish homeostasis. The additive effect of AZT on these pathways in the presence of SMM suggests that the two treatments activate the pathways by separate mechanisms. In the presence of SMM, these pathways are likely up-regulated by a pathway involving ERK1/2 phosphorylation of SREBP [50], [51]. Although it is not clear how AZT might be acting, certain hypothesis can be put forward. In cells arrested at G2/M, for example, the transcriptional and DNA-binding activity of SREBP-1 is determined by hyper-phosphorylation mediated by CDK-1/cyclin B [50]. These previous observations suggest a possible link between AZT-induced inhibition of cell cycle genes, an additional finding in the present study (Fig. 2 and Supporting Information, Table S5), and the lipid/cholesterol pathways. In addition, AZT and another macrolide, erythromycin, can directly interact with phospholipids, modify membrane biophysical properties, and affect membrane dynamics in living cells [52]–[54], processes that have been hypothesized account for activity and/or toxicity. These findings are also likely relevant to the mechanism by which AZT induces phospholipidosis, a well-described *in vivo* effect of AZT [55]. Additionally, given that one essential function of SREBP appears to be its ability to monitor cell membrane composition and to adjust lipid synthesis accordingly [56], it is possible that the interaction of macrolides with the membrane provides a strong signal to the cell to compensate for membrane disruptions. Given all of these diverse findings, it is reasonable to propose a link between the membrane accumulation of AZT, the induction of phospholipidosis by AZT, the up-regulation of lipid gene metabolism reported here, and the final clinical outcome.

Finally, down-regulation of mitosis/cell cycle genes by AZT could also contribute to its therapeutic effects. For example, a decrease in the proliferation of airway epithelial cells, such as mucus-producing cells, in response to luminal bacterial and inflammatory stimuli might result in reduced inflammatory burden by preventing the expression of these cells. Studies in cancer cells or virally-transformed cells showed no effect of macrolide treatment on proliferation, viability or distribution of the cell cycle phase [57], [58]. In contrast, other studies using a cell system similar to ours, showed that clarithromycin delayed progression of primary bronchial cells from G1 to S phase [59]. A reduction in the expression of cell cycle genes was not considered in the latter study, but a correlation to ERK1/2 activation, a well-established pathway for controlling the cell cycle, was described [59]. In fact, regulation of the ERK1/2 signaling pathway has been proposed as a unifying mechanism explaining the complex immunomodulation of macrolides (reviewed in [3]). Studies using the macrolide clarythromycin demonstrated decreased, and then increased, phosphorylation of ERK in response to a bacterial stimulant, flagellin, at 9 and 24 hrs, respectively [60]. A similar finding involving reduction in ERK phosphorylation after LPS stimulation in the presence of macrolide antibiotics has recently been reported by a separate group [36], and it has been hypothesized that reduction in ERK activation could lead to reduced mucin gene expression. Although our studies did not evaluate ERK phosphorylation, they are consistent with this hypothesis. On the other hand, based upon studies demonstrating increased transcriptional activation of SREBP-target genes after ERK-mediated phosphorylation of SREBP [50], [51], a reduction in ERK phosphorylation does not help to explain the up-regulation of lipid/fatty acid/cholesterol metabolic pathways observed in our studies.

In summary, global gene expression data suggest that the macrolide AZT can alter inflammatory responses as predicted from clinical observations, but these responses are highly variable and gene-specific, with an overall tendency toward global down-regulation. The striking down-regulation of the RNA of specific genes, e.g., MUC5AC and MMP9, is likely to be highly relevant to clinical outcomes, and future studies focusing on the role of these individual effects should help identify the dominant pathways involved in the clinical actions of AZT. The increased IL-8 protein secretion after inflammatory challenge in the presence of AZT points to the complexity of interpreting gene expression data in light of the inflammatory outcome. The remarkable and highly significant up-regulation of lipid/cholesterol metabolic pathways and down-regulation of cell cycle/mitosis genes is worthy of consideration in future studies evaluating the mode of action of macrolides.

## Supporting Information

Methods S1 (0.05 MB DOC)

Table S1 (0.80 MB XLS)

Table S2 (0.84 MB RTF)

Table S3 (0.58 MB DOC)

Table S4 (0.09 MB DOC)

Table S5 (0.11 MB DOC)

Table S6 (0.53 MB DOC)

## References

[1] Jaffe A, Bush A (2001). Anti-inflammatory effects of macrolides in lung disease.. Pediatr Pulmonol.

[2] Pechere JC (2001). New perspectives on macrolide antibiotics.. Int J Antimicrob Agents.

[3] Shinkai M, Henke MO, Rubin BK (2008). Macrolide antibiotics as immunomodulatory medications: proposed mechanisms of action.. Pharmacol Ther.

[4] Wagner T, Soong G, Sokol S, Saiman L, Prince A (2005). Effects of azithromycin on clinical isolates of Pseudomonas aeruginosa from cystic fibrosis patients.. Chest.

[5] Hoffmann N, Lee B, Hentzer M, Rasmussen TB, Song Z (2007). Azithromycin blocks quorum sensing and alginate polymer formation and increases the sensitivity to serum and stationary-growth-phase killing of Pseudomonas aeruginosa and attenuates chronic P. aeruginosa lung infection in Cftr(-/-) mice.. Antimicrob Agents Chemother.

[6] Sakito O, Kadota J, Kohno S, Abe K, Shirai R (1996). Interleukin 1 beta, tumor necrosis factor alpha, and interleukin 8 in bronchoalveolar lavage fluid of patients with diffuse panbronchiolitis: a potential mechanism of macrolide therapy.. Respiration.

[7] Tsai WC, Rodriguez ML, Young KS, Deng JC, Thannickal VJ (2004). Azithromycin blocks neutrophil recruitment in Pseudomonas endobronchial infection.. Am J Respir Crit Care Med.

[8] Khair OA, Devalia JL, Abdelaziz MM, Sapsford RJ, Davies RJ (1995). Effect of erythromycin on Haemophilus influenzae endotoxin-induced release of IL-6, IL-8 and sICAM-1 by cultured human bronchial epithelial cells.. Eur Respir J.

[9] Takizawa H, Desaki M, Ohtoshi T, Kawasaki S, Kohyama T (1997). Erythromycin modulates IL-8 expression in normal and inflamed human bronchial epithelial cells.. Am J Respir Crit Care Med.

[10] Kawasaki S, Takizawa H, Ohtoshi T, Takeuchi N, Kohyama T (1998). Roxithromycin inhibits cytokine production by and neutrophil attachment to human bronchial epithelial cells in vitro.. Antimicrob Agents Chemother.

[11] Kadota J, Sakito O, Kohno S, Sawa H, Mukae H (1993). A mechanism of erythromycin treatment in patients with diffuse panbronchiolitis.. Am Rev Respir Dis.

[12] Oda H, Kadota J, Kohno S, Hara K (1994). Erythromycin inhibits neutrophil chemotaxis in bronchoalveoli of diffuse panbronchiolitis.. Chest.

[13] Ichikawa Y, Ninomiya H, Koga H, Tanaka M, Kinoshita M (1992). Erythromycin reduces neutrophils and neutrophil-derived elastolytic-like activity in the lower respiratory tract of bronchiolitis patients.. Am Rev Respir Dis.

[14] Yamasawa H, Oshikawa K, Ohno S, Sugiyama Y (2004). Macrolides inhibit epithelial cell-mediated neutrophil survival by modulating granulocyte macrophage colony-stimulating factor release.. Am J Respir Cell Mol Biol.

[15] Koch CC, Esteban DJ, Chin AC, Olson ME, Read RR (2000). Apoptosis, oxidative metabolism and interleukin-8 production in human neutrophils exposed to azithromycin: effects of Streptococcus pneumoniae.. J Antimicrob Chemother.

[16] Culic O, Erakovic V, Cepelak I, Barisic K, Brajsa K (2002). Azithromycin modulates neutrophil function and circulating inflammatory mediators in healthy human subjects.. Eur J Pharmacol.

[17] Hodge S, Hodge G, Brozyna S, Jersmann H, Holmes M (2006). Azithromycin increases phagocytosis of apoptotic bronchial epithelial cells by alveolar macrophages.. Eur Respir J.

[18] Mankin AS (2008). Macrolide myths.. Curr Opin Microbiol.

[19] Imamura Y, Yanagihara K, Mizuta Y, Seki M, Ohno H (2004). Azithromycin inhibits MUC5AC production induced by the Pseudomonas aeruginosa autoinducer N-(3-Oxododecanoyl) homoserine lactone in NCI-H292 Cells.. Antimicrob Agents Chemother.

[20] Shimizu T, Shimizu S, Hattori R, Gabazza EC, Majima Y (2003). In vivo and in vitro effects of macrolide antibiotics on mucus secretion in airway epithelial cells.. Am J Respir Crit Care Med.

[21] Kanai K, Asano K, Hisamitsu T, Suzaki H (2004). Suppression of matrix metalloproteinase production from nasal fibroblasts by macrolide antibiotics in vitro.. Eur Respir J.

[22] Hashimoto N, Kawabe T, Hara T, Imaizumi K, Wakayama H (2001). Effect of erythromycin on matrix metalloproteinase-9 and cell migration.. J Lab Clin Med.

[23] Ribeiro CMP, Paradiso AM, Carew MA, Shears SB, Boucher RC (2005). Cystic fibrosis airway epithelial Ca2+i signaling. The mechanism for the larger agonist-mediated Ca2+i signals in human cystic fibrosis airway epithelia.. J Biol Chem.

[24] Ribeiro CMP, Paradiso AM, Schwab U, Perez-Vilar J, Jones L (2005). Chronic airway infection/Inflammation Induces a Ca2+i-dependent hyperinflammatory response in human cystic fibrosis airway epithelia.. J Biol Chem.

[25] Wilms EB, Touw DJ, Heijerman HG (2006). Pharmacokinetics of azithromycin in plasma, blood, polymorphonuclear neutrophils and sputum during long-term therapy in patients with cystic fibrosis.. Ther Drug Monit.

[26] Irizarry RA, Hobbs B, Collin F, Beazer-Barclay YD, Antonellis KJ (2003). Exploration, normalization, and summaries of high density oligonucleotide array probe level data.. Biostatistics.

[27] Pfaffl MW (2001). A new mathematical model for relative quantification in real-time RT-PCR.. Nucleic Acids Res.

[28] White NM, Corey DA, Kelley TJ (2004). Mechanistic similarities between cultured cell models of cystic fibrosis and niemann-pick type C.. Am J Respir Cell Mol Biol.

[29] Ribeiro CM, Paradiso AM, Livraghi A, Boucher RC (2003). The mitochondrial barriers segregate agonist-induced calcium-dependent functions in human airway epithelia.. J Gen Physiol.

[30] Kurdowska A, Noble JM, Griffith DE (2001). The effect of azithromycin and clarithromycin on ex vivo interleukin-8 (IL-8) release from whole blood and IL-8 production by human alveolar macrophages.. J Antimicrob Chemother.

[31] Shinkai M, Foster GH, Rubin BK (2006). Macrolide antibiotics modulate ERK phosphorylation and IL-8 and GM-CSF production by human bronchial epithelial cells.. Am J Physiol.

[32] Parnham MJ, Culic O, Erakovic V, Munic V, Popovic-Grle S (2005). Modulation of neutrophil and inflammation markers in chronic obstructive pulmonary disease by short-term azithromycin treatment.. Eur J Pharmacol.

[33] Verleden GM, Vanaudenaerde BM, Dupont LJ, Van Raemdonck DE (2006). Azithromycin reduces airway neutrophilia and interleukin-8 in patients with bronchiolitis obliterans syndrome.. Am J Respir Crit Care Med.

[34] Janowski BA (2002). The hypocholesterolemic agent LY295427 upregulates INSIG-1, identifying the INSIG-1 protein as a mediator of cholesterol homeostasis through SREBP.. Proc Natl Acad Sci U S A.

[35] Kanai K, Asano K, Hisamitsu T, Suzaki H (2004). Suppression of matrix metalloproteinase-9 production from neutrophils by a macrolide antibiotic, roxithromycin, in vitro.. Mediators Inflamm.

[36] Ishimoto H, Mukae H, Sakamoto N, Amenomori M, Kitazaki T (2009). Different effects of telithromycin on MUC5AC production induced by human neutrophil peptide-1 or lipopolysaccharide in NCI-H292 cells compared with azithromycin and clarithromycin.. J Antimicrob Chemother.

[37] Tamaoki J, Takeyama K, Tagaya E, Konno K (1995). Effect of clarithromycin on sputum production and its rheological properties in chronic respiratory tract infections.. Antimicrob Agents Chemother.

[38] Van den Steen PE, Proost P, Wuyts A, Van Damme J, Opdenakker G (2000). Neutrophil gelatinase B potentiates interleukin-8 tenfold by aminoterminal processing, whereas it degrades CTAP-III, PF-4, and GRO-alpha and leaves RANTES and MCP-2 intact.. Blood.

[39] Yu Q, Stamenkovic I (2000). Cell surface-localized matrix metalloproteinase-9 proteolytically activates TGF-beta and promotes tumor invasion and angiogenesis.. Genes Dev.

[40] Kelly EA, Busse WW, Jarjour NN (2000). Increased matrix metalloproteinase-9 in the airway after allergen challenge.. Am J Respir Crit Care Med.

[41] Gaggar A, Li Y, Weathington N, Winkler M, Kong M (2007). Matrix metalloprotease-9 dysregulation in lower airway secretions of cystic fibrosis patients.. Am J Physiol Lung Cell Mol Physiol.

[42] Sagel SD, Kapsner RK, Osberg I (2005). Induced sputum matrix metalloproteinase-9 correlates with lung function and airway inflammation in children with cystic fibrosis.. Pediatr Pulmonol.

[43] Askew DJ, Silverman GA (2008). Intracellular and extracellular serpins modulate lung disease.. J Perinatol.

[44] Liu K, Xu Q (2008). Roxithromycin inhibits the effector phase of delayed-type hypersensitivity.. Int Immunopharmacol.

[45] Simpson JL, Powell H, Boyle MJ, Scott RJ, Gibson PG (2008). Clarithromycin targets neutrophilic airway inflammation in refractory asthma.. Am J Respir Crit Care Med.

[46] Ogawa M, Suzuki J, Takayama K, Isobe M (2009). Matrix metalloproteinase suppression induced by clarithromycin in murine cardiac allografts.. Transplant Proc.

[47] Maxfield FR, Tabas I (2005). Role of cholesterol and lipid organization in disease.. Nature.

[48] D'Avila H, Maya-Monteiro CM, Bozza PT (2008). Lipid bodies in innate immune response to bacterial and parasite infections.. Int Immunopharmacol.

[49] Van Bambeke F, Gerbaux C, Michot JM, d'Yvoire MB, Montenez JP (1998). Lysosomal alterations induced in cultured rat fibroblasts by long-term exposure to low concentrations of azithromycin.. J Antimicrob Chemother.

[50] Bengoechea-Alonso MT, Punga T, Ericsson J (2005). Hyperphosphorylation regulates the activity of SREBP1 during mitosis.. Proc Natl Acad Sci U S A.

[51] Arito M, Horiba T, Hachimura S, Inoue J, Sato R (2008). Growth factor-induced phosphorylation of sterol regulatory element-binding proteins inhibits sumoylation, thereby stimulating the expression of their target genes, low density lipoprotein uptake, and lipid synthesis.. J Biol Chem.

[52] Tyteca D, Schanck A, Dufrene YF, Deleu M, Courtoy PJ (2003). The macrolide antibiotic azithromycin interacts with lipids and affects membrane organization and fluidity: studies on Langmuir-Blodgett monolayers, liposomes and J774 macrophages.. J Membr Biol.

[53] Fa N, Lins L, Courtoy PJ, Dufrene Y, Van Der SP (2007). Decrease of elastic moduli of DOPC bilayers induced by a macrolide antibiotic, azithromycin.. Biochim Biophys Acta.

[54] Matsumori N, Morooka A, Murata M (2006). Detailed description of the conformation and location of membrane-bound erythromycin a using isotropic bicelles.. J Med Chem.

[55] Baronas ET, Lee JW, Alden C, Hsieh FY (2007). Biomarkers to monitor drug-induced phospholipidosis.. Toxicol Appl Pharmacol.

[56] Dobrosotskaya IY, Seegmiller AC, Brown MS, Goldstein JL, Rawson RB (2002). Regulation of SREBP processing and membrane lipid production by phospholipids in Drosophila.. Science.

[57] Abe S, Nakamura H, Inoue S, Takeda H, Saito H (2000). Interleukin-8 gene repression by clarithromycin is mediated by the activator protein-1 binding site in human bronchial epithelial cells.. Am J Respir Cell Mol Biol.

[58] Chen SZ, Jiang M, Zhen YS (2005). HERG K+ channel expression-related chemosensitivity in cancer cells and its modulation by erythromycin.. Cancer Chemother Pharmacol.

[59] Shinkai M, Tamaoki J, Kobayashi H, Kanoh S, Motoyoshi K (2006). Clarithromycin delays progression of bronchial epithelial cells from G1 phase to S phase and delays cell growth via extracellular signal-regulated protein kinase suppression.. Antimicrob Agents Chemother.

[60] Shinkai M, Lopez-Boado YS, Rubin BK (2007). Clarithromycin has an immunomodulatory effect on ERK-mediated inflammation induced by Pseudomonas aeruginosa flagellin.. J Antimicrob Chemother.

