# Supplementary material for: Azithromycin Treatment Alters Gene Expression in Inflammatory, Lipid Metabolism, and Cell Cycle Pathways in Well-Differentiated Human Airway Epithelia
Source: PLoS One. 2009 Jun 5;4(6):e5806. doi: 10.1371/journal.pone.0005806 (PMC2688381; doi:10.1371/journal.pone.0005806)
Supplement: Methods S1 — (0.05 MB DOC) [file pone.0005806.s001.doc]

**Supporting Information.**

**Methods S1**

**Statistical Analysis of Microarrays:** For our statistical analysis of the Affymetrix chip results, we first estimated expression levels according to the method of GCRMA [1] using the parameters provided by just GCRMA (such as optical correction and quantile normalization) applied to 40 chips derived from the hybridization of four individual cell donors and the 10 possible treatments as described in the Methods. Using Bioconductor RNA digestion plots, we eliminated 6 chips having slopes that deviated excessively from the median slopes, thus producing the set of 34 chips that passed the diagnostic check. We then re-ran GCRMA to obtain the estimated expression values for on the final set of 34 chips. The final data set is represented by the following schematic, where the four individual codes are labeled A, B, C, and D, and the conditions tested (specified in Table 1) are labeled 1-10 (it is worthwhile to note that at least 3 chips survived quality control for each of the 10 treatments):

| A1 | A2 | A3 |  | A5 | A6 | A7 | A8 | A9 | A10 |
| --- | --- | --- | --- | --- | --- | --- | --- | --- | --- |
| B1 | B2 | B3 | B4 | B5 | B6 | B7 | B8 | B9 | B10 |
|  | C2 | C3 | C4 | C5 |  | C7 |  | C9 | C10 |
| D1 | D2 | D3 | D4 | D5 | D6 | D7 | D8 |  |  |

Analysis of the high quality chips followed two main directions, paired comparisons and unsupervised clustering. The local-pooled-error (LPE) method [2] was applied to each of the pairs selected for analysis (these pairs are indicated in Table1 in the text). In the analysis of treatment pairs, one is considered control and the other treatment. For each probeset, the LPE procedure produced a z-score, the associated p-value (z.pv), a false discovery rate (FDR) value determined by resampling, and the mean difference. The resampling method, described in the Bioconductor LPE vignette [3], used five iterations.

For our analysis, the gene (probeset) expression differences between pairs were ranked by z-score, and this ranked list was used to determine gene ontology (GO) groups that were over-represented in differentially regulated genes between the treatment pairs. Expression levels and differences were computed for each probeset, whereas in the GO analysis, hypergeometric probabilities were computed on the basis of gene presence in a list of probesets. We used the MultiGOMatrix (developed by Harry Hurd) to get a quick assessment of the most significantly present GO groups represented in the differences of a pair. This method displays the hypergeometric probabilities for every significant GO group found in a nested collection of probesets defined by different threshold levels of a sample selection parameter, such as FDR, or z-score p-value. This method allowed for a global overview of the effect that a treatment had on a GO group and avoids the problem of having to guess a single number of genes that goes into the analysis, a number that may be too conservative or too liberal to detect the most significant findings. In these studies, for a fixed comparison pair, we ranked the probesets according to z-score and the nested collection of probesets were defined simply by using the TOPN (top number) probesets for TOPN=20, 40, 80, 160, 320, 640. All the MGM matrices using the TOPN method of defining the probe sets are available in the supplemental data (Supplemental Data, Results, Section 1). Additional details about the MGM matrices is available in that section.

Whereas analysis of pairs was used to detect strong differences between probesets of a treatment pair, hierarchical clustering was used visualize global gene changes and relationships among treatments. For this work, we limited the number of probesets to those identified as the most strongly differentiated in the paired comparisons. For each probeset, the vector to be clustered was the mean (over the good chips) of GCRMA expression levels. Clusters were computed using the correlation metric and complete linkage. In this paper, the collection of probesets to be clustered was the union over all the comparison pairs and probesets for which the FDR was no larger than 0.1.

**References**
