# Supplementary material for: Azithromycin Treatment Alters Gene Expression in Inflammatory, Lipid Metabolism, and Cell Cycle Pathways in Well-Differentiated Human Airway Epithelia
Source: PLoS One. 2009 Jun 5;4(6):e5806. doi: 10.1371/journal.pone.0005806 (PMC2688381; doi:10.1371/journal.pone.0005806)
Supplement: Table S3 — (0.58 MB DOC) [file pone.0005806.s004.doc]

#### Supporting Information.

Table S3. Regulation (up or down) of inflammation-related genes.

| **Gene Title** | **Gene Symbol** | **AZT 6 vs PBS6** | **AZT24 vs PBS24** | **SMM6 vs PBS6** | **SMM24 vs PBS24** | **AZT48 SMM6vs SMM6** | **AZT72 SMM24 vs SMM24** |
| --- | --- | --- | --- | --- | --- | --- | --- |
| ADAM metallopeptidase domain 15 (metargidin) | ADAM15 |  |  | UP |  | DOWN | DOWN |
| ADAM metallopeptidase domain 28 | ADAM28 |  |  | UP |  |  |  |
| ADAM metallopeptidase with thrombospondin type 1 motif, 1 | ADAMTS1 |  |  | UP |  | DOWN | DOWN |
| adrenomedullin | ADM | UP | UP | UP | UP |  |  |
| adrenergic, beta-2-, receptor, surface | ADRB2 |  |  | UP | UP |  | DOWN |
| arachidonate 15-lipoxygenase | ALOX15 |  |  |  | DOWN |  |  |
| arachidonate 5-lipoxygenase | ALOX5 |  |  |  |  | UP |  |
| adhesion molecule with Ig-like domain 2 | AMIGO2 |  | DOWN |  |  | DOWN | DOWN |
| angiopoietin-like 4 | ANGPTL4 |  |  | UP | UP | DOWN |  |
| adaptor-related protein complex 2, beta 1 subunit | AP2B1 |  |  |  | UP |  |  |
| amphiregulin (schwannoma-derived growth factor) /// similar to Amphiregulin precursor (AR) (Colorectum cell-derived growth factor) (CRDGF) | AREG /// LOC727738 | UP | DOWN | UP | UP |  |  |
| ADP-ribosylation factor 4 | ARF4 |  |  |  | UP |  |  |
| arginase, type II | ARG2 |  |  | UP |  |  |  |
| Rho GDP dissociation inhibitor (GDI) alpha | ARHGDIA |  |  | UP |  | DOWN |  |
| rho/rac guanine nucleotide exchange factor (GEF) 2 | ARHGEF2 |  |  | UP |  |  |  |
| artemin | ARTN |  | UP |  |  |  |  |
| type 1 tumor necrosis factor receptor shedding aminopeptidase regulator | ARTS-1 |  | DOWN |  |  | UP |  |
| ATPase inhibitory factor 1 | ATPIF1 |  |  | DOWN |  |  |  |
| alpha-2-glycoprotein 1, zinc-binding | AZGP1 |  |  |  |  | DOWN | DOWN |
| B-cell receptor-associated protein 29 | BCAP29 |  |  | UP |  |  |  |
| B-cell CLL/lymphoma 10 | BCL10 |  |  | UP |  |  |  |
| BCL2-related protein A1 | BCL2A1 |  | DOWN | UP | UP |  | DOWN |
| B-cell CLL/lymphoma 3 | BCL3 |  |  | UP | UP |  |  |
| BH3 interacting domain death agonist | BID |  |  | UP |  |  |  |
| BCL2-interacting killer (apoptosis-inducing) | BIK |  |  | UP |  |  |  |
| baculoviral IAP repeat-containing 3 | BIRC3 |  |  | UP | UP |  |  |
| baculoviral IAP repeat-containing 5 (survivin) | BIRC5 |  | DOWN |  | DOWN |  |  |
| B-cell linker | BLNK |  | UP |  |  | UP |  |
| bone morphogenetic protein 2 | BMP2 |  |  | UP |  |  |  |
| BCL2/adenovirus E1B 19kDa interacting protein 3 | BNIP3 |  |  |  | UP |  |  |
| BUB1 budding uninhibited by benzimidazoles 1 homolog beta (yeast) | BUB1B |  | DOWN |  |  | DOWN |  |
| complement component 1, s subcomponent | C1S |  |  |  | UP |  | UP |
| chromosome 20 open reading frame 42 | C20orf42 |  |  | UP |  | DOWN | DOWN |
| carbonic anhydrase XII | CA12 |  |  | UP | UP |  |  |
| Calreticulin | CALR |  | DOWN |  | UP | UP |  |
| calcium/calmodulin-dependent protein kinase ID | CAMK1D |  |  |  | DOWN |  |  |
| calcium/calmodulin-dependent protein kinase II inhibitor 1 | CAMK2N1 |  |  |  |  | DOWN |  |
| calpain 13 | CAPN13 |  |  |  | UP |  |  |
| caspase 10, apoptosis-related cysteine peptidase | CASP10 |  | UP |  |  |  | UP |
| chromobox homolog 4 (Pc class homolog, Drosophila) | CBX4 |  |  | UP | UP | DOWN |  |
| chemokine (C-C motif) ligand 2 | CCL2 |  |  |  |  | UP |  |
| chemokine (C-C motif) ligand 20 | CCL20 | UP | DOWN | UP | UP |  | DOWN |
| CD24 molecule | CD24 |  |  |  | DOWN |  | UP |
| CD44 molecule (Indian blood group) /// mitogen-activated protein kinase 10 | CD44 /// MAPK10 |  |  | UP |  |  |  |
| CD46 molecule, complement regulatory protein | CD46 |  |  |  | UP |  |  |
| CD55 molecule, decay accelerating factor for complement (Cromer blood group) | CD55 |  |  | UP | UP | DOWN | DOWN |
| CD74 molecule, major histocompatibility complex, class II invariant chain | CD74 |  |  |  | UP |  |  |
| CD99 molecule | CD99 |  |  |  | UP | DOWN |  |
| cadherin 6, type 2, K-cadherin (fetal kidney) | CDH6 |  | DOWN |  |  |  | DOWN |
| cyclin-dependent kinase inhibitor 1A (p21, Cip1) | CDKN1A |  | UP |  |  |  | UP |
| carcinoembryonic antigen-related cell adhesion molecule 1 (biliary glycoprotein) | CEACAM1 |  | UP | UP | UP |  |  |
| CCAAT/enhancer binding protein (C/EBP), beta | CEBPB |  |  | UP |  |  |  |
| complement factor B | CFB |  |  | UP | UP |  |  |
| CASP8 and FADD-like apoptosis regulator | CFLAR |  |  | UP |  |  |  |
| carbohydrate (N-acetylglucosamine-6-O) sulfotransferase 2 | CHST2 |  |  |  |  | UP |  |
| carbohydrate (N-acetylglucosamine 6-O) sulfotransferase 4 | CHST4 |  |  | UP |  |  |  |
| claudin 1 | CLDN1 | UP |  | UP |  |  |  |
| claudin 10 | CLDN10 |  | DOWN |  | UP | DOWN | DOWN |
| C-type lectin domain family 2, member B /// CMT1A duplicated region transcript 15 pseudogene | CLEC2B /// CDRT15P |  | UP |  |  | UP |  |
| C-type lectin domain family 7, member A | CLEC7A |  | UP |  | DOWN | UP | UP |
| clusterin | CLU |  |  |  |  | DOWN | DOWN |
| CKLF-like MARVEL transmembrane domain containing 4 | CMTM4 | UP |  |  |  |  |  |
| Contactin 1 | CNTN1 |  |  |  |  | UP |  |
| contactin associated protein-like 3 /// contactin associated protein-like 3B /// similar to Contactin-associated protein-like 3 precursor (Cell recognition molecule Caspr3) | CNTNAP3 /// CNTNAP3B /// LOC728577 |  |  |  |  |  | DOWN |
| collagen, type XII, alpha 1 | COL12A1 |  | DOWN |  |  | DOWN | DOWN |
| collagen, type IV, alpha 6 | COL4A6 |  |  |  |  |  | DOWN |
| carboxypeptidase D | CPD |  |  |  |  |  | UP |
| carboxypeptidase E | CPE |  | DOWN |  |  |  |  |
| carboxypeptidase X (M14 family), member 2 | CPXM2 |  | UP |  |  | UP | UP |
| cysteine-rich protein 1 (intestinal) /// galactokinase 2 | CRIP1 /// GALK2 |  |  |  |  | DOWN | DOWN |
| cytokine receptor-like factor 1 | CRLF1 |  |  |  |  | DOWN | DOWN |
| colony stimulating factor 3 (granulocyte) | CSF3 |  |  | UP | UP |  | DOWN |
| connective tissue growth factor | CTGF |  | DOWN | UP |  | DOWN |  |
| catenin (cadherin-associated protein), alpha-like 1 | CTNNAL1 |  |  |  |  |  | DOWN |
| cathepsin A | CTSA |  | UP |  |  | UP | UP |
| cathepsin B | CTSB |  |  |  |  |  |  |
| cathepsin H | CTSH |  |  |  |  |  | UP |
| chemokine (C-X-C motif) ligand 1 (melanoma growth stimulating activity, alpha) | CXCL1 |  |  | UP | UP |  |  |
| chemokine (C-X-C motif) ligand 14 | CXCL14 |  |  |  |  |  | DOWN |
| chemokine (C-X-C motif) ligand 16 | CXCL16 |  |  |  | UP |  |  |
| chemokine (C-X-C motif) ligand 2 | CXCL2 |  |  | UP | UP |  |  |
| chemokine (C-X-C motif) ligand 3 | CXCL3 |  | DOWN | UP | UP |  | DOWN |
| chemokine (C-X-C motif) ligand 5 | CXCL5 |  |  | UP | UP |  |  |
| chemokine (C-X-C motif) ligand 6 (granulocyte chemotactic protein 2) | CXCL6 |  | DOWN | UP | UP | DOWN |  |
| chemokine (C-X-C motif) receptor 7 | CXCR7 | UP |  | UP |  |  |  |
| cytochrome P450, family 26, subfamily B, polypeptide 1 | CYP26B1 |  |  | UP | UP |  |  |
| D site of albumin promoter (albumin D-box) binding protein | DBP |  | UP |  |  |  |  |
| doublecortin domain containing 2 | DCDC2 | DOWN |  |  |  |  |  |
| DEAD (Asp-Glu-Ala-Asp) box polypeptide 58 | DDX58 | UP |  |  |  | UP |  |
| defensin, beta 1 | DEFB1 |  |  |  | UP | UP |  |
| defensin, beta 4 /// similar to Beta-defensin 2 precursor (BD-2) (hBD-2) (Defensin, beta 2) (Skin-antimicrobial peptide 1) (SAP1) | DEFB4 /// LOC728454 |  |  | UP | UP |  | DOWN |
| DnaJ (Hsp40) related, subfamily B, member 13 | DNAJB13 |  |  |  |  | DOWN |  |
| DnaJ (Hsp40) homolog, subfamily B, member 9 | DNAJB9 |  |  | UP | UP |  |  |
| DnaJ (Hsp40) homolog, subfamily C, member 10 | DNAJC10 |  |  |  | UP |  |  |
| DnaJ (Hsp40) homolog, subfamily C, member 12 | DNAJC12 |  |  | UP | UP |  |  |
| desmocollin 2 | DSC2 |  |  | UP |  |  |  |
| desmocollin 3 | DSC3 |  | DOWN |  |  | DOWN | DOWN |
| dual oxidase 2 | DUOX2 |  |  | UP | UP | DOWN |  |
| dual oxidase maturation factor 2 | DUOXA2 |  |  | UP | UP | DOWN | DOWN |
| dual specificity phosphatase 1 | DUSP1 |  |  | UP |  |  |  |
| dual specificity phosphatase 5 | DUSP5 | UP | UP | UP | UP |  |  |
| dual specificity phosphatase 6 | DUSP6 |  |  | UP | UP |  |  |
| Endothelin converting enzyme 1 | ECE1 |  |  | UP |  |  |  |
| endothelial cell growth factor 1 (platelet-derived) | ECGF1 |  |  |  | UP |  |  |
| extracellular matrix protein 1 | ECM1 |  |  |  | UP |  |  |
| epithelial cell transforming sequence 2 oncogene | ECT2 |  | DOWN |  |  |  |  |
| ER degradation enhancer, mannosidase alpha-like 1 | EDEM1 |  |  | UP |  |  |  |
| endothelial differentiation, lysophosphatidic acid G-protein-coupled receptor, 2 | EDG2 |  |  | UP | UP |  |  |
| EGF-like repeats and discoidin I-like domains 3 | EDIL3 |  | UP |  |  | UP |  |
| EF-hand domain (C-terminal) containing 1 | EFHC1 |  |  |  | DOWN | DOWN |  |
| ephrin-A1 | EFNA1 |  |  | UP | UP |  |  |
| Epidermal growth factor receptor (erythroblastic leukemia viral (v-erb-b) oncogene homolog, avian) | EGFR | DOWN | UP | DOWN |  |  |  |
| egl nine homolog 3 (C. elegans) | EGLN3 | UP |  | UP | UP |  |  |
| eukaryotic translation initiation factor 5A | EIF5A | UP |  | UP |  | UP | DOWN |
| epithelial membrane protein 1 | EMP1 |  |  | UP | UP | DOWN |  |
| epoxide hydrolase 1, microsomal (xenobiotic) | EPHX1 |  |  |  | DOWN |  |  |
| epiregulin | EREG |  |  | UP | UP |  |  |
| ERO1-like (S. cerevisiae) | ERO1L |  |  | UP | UP |  |  |
| ERBB receptor feedback inhibitor 1 | ERRFI1 |  |  | UP | UP |  |  |
| v-ets erythroblastosis virus E26 oncogene homolog 1 (avian) | ETS1 |  |  | UP |  |  |  |
| coagulation factor III (thromboplastin, tissue factor) | F3 |  | DOWN |  | UP | DOWN |  |
| Fas (TNF receptor superfamily, member 6) | FAS |  |  | UP |  |  |  |
| F-box protein 2 | FBXO2 |  |  |  |  | UP |  |
| Fc fragment of IgG binding protein | FCGBP |  |  |  | UP |  | DOWN |
| fasciculation and elongation protein zeta 1 (zygin I) | FEZ1 |  |  |  |  |  | DOWN |
| fibroblast growth factor receptor 3 (achondroplasia, thanatophoric dwarfism) | FGFR3 |  |  | UP |  | DOWN | DOWN |
| FK506 binding protein 11, 19 kDa | FKBP11 |  | DOWN |  | UP | DOWN |  |
| FK506 binding protein 1A, 12kDa | FKBP1A |  |  |  |  | DOWN |  |
| filamin A, alpha (actin binding protein 280) | FLNA |  |  |  | UP |  |  |
| fibronectin leucine rich transmembrane protein 2 | FLRT2 |  | DOWN |  |  | DOWN |  |
| fibronectin leucine rich transmembrane protein 3 | FLRT3 |  | DOWN |  | UP |  | DOWN |
| fibronectin 1 | FN1 |  | DOWN |  | DOWN |  | UP |
| v-fos FBJ murine osteosarcoma viral oncogene homolog | FOS | UP |  | UP | UP | DOWN |  |
| FOS-like antigen 1 | FOSL1 |  |  | UP |  | DOWN |  |
| FOS-like antigen 2 | FOSL2 |  |  | UP |  |  |  |
| ferritin, heavy polypeptide 1 | FTH1 |  | UP |  | UP |  |  |
| ferritin, light polypeptide | FTL |  | UP |  |  | UP | UP |
| fusion (involved in t(12;16) in malignant liposarcoma) | FUS |  |  |  |  | DOWN |  |
| fucosyltransferase 11 (alpha (1,3) fucosyltransferase) | FUT11 |  |  | UP |  |  |  |
| fucosyltransferase 2 (secretor status included) | FUT2 |  |  | UP |  | DOWN |  |
| fucosyltransferase 3 (galactoside 3(4)-L-fucosyltransferase, Lewis blood group) | FUT3 |  |  | UP | UP |  |  |
| fucosyltransferase 6 (alpha (1,3) fucosyltransferase) | FUT6 |  |  | UP | UP |  |  |
| FYN binding protein (FYB-120/130) | FYB |  |  | DOWN |  | UP |  |
| GATA binding protein 3 | GATA3 |  |  |  | UP | DOWN |  |
| guanylate binding protein 1, interferon-inducible, 67kDa /// guanylate binding protein 1, interferon-inducible, 67kDa | GBP1 |  |  | UP |  |  |  |
| guanylate binding protein 2, interferon-inducible /// guanylate binding protein 2, interferon-inducible | GBP2 |  |  |  |  | UP |  |
| guanylate binding protein 3 | GBP3 |  |  |  |  | UP |  |
| GTP cyclohydrolase 1 (dopa-responsive dystonia) | GCH1 |  |  | UP | UP |  |  |
| growth differentiation factor 15 | GDF15 |  | UP | UP | UP | UP | UP |
| gap junction protein, alpha 1, 43kDa (connexin 43) | GJA1 |  | UP |  |  | UP |  |
| gap junction protein, beta 6 (connexin 30) | GJB6 |  | DOWN |  | UP | DOWN | DOWN |
| glycoprotein (transmembrane) nmb | GPNMB |  | UP | DOWN |  | UP | UP |
| glutathione S-transferase A3 | GSTA3 |  |  |  | DOWN | DOWN | DOWN |
| GULP, engulfment adaptor PTB domain containing 1 | GULP1 |  | UP |  | DOWN | UP | UP |
| hemoglobin, beta /// hemoglobin, beta | HBB |  |  |  |  | DOWN | DOWN |
| heparin-binding EGF-like growth factor | HBEGF |  |  | UP |  |  |  |
| histone deacetylase 9 | HDAC9 |  |  | UP |  |  |  |
| Helicase, lymphoid-specific | HELLS |  | DOWN |  |  |  |  |
| hypoxia-inducible factor 1, alpha subunit (basic helix-loop-helix transcription factor) | HIF1A |  |  |  |  | UP | UP |
| hypoxia-inducible protein 2 | HIG2 |  |  | UP | UP |  |  |
| Homeodomain interacting protein kinase 2 | HIPK2 |  |  | UP |  |  |  |
| histone cluster 1, H2bc | HIST1H2BC |  |  |  |  | DOWN |  |
| histone cluster 1, H2bg | HIST1H2BG | DOWN |  | DOWN |  | DOWN |  |
| major histocompatibility complex, class II, DP beta 1 | HLA-DPB1 |  |  |  |  |  |  |
| major histocompatibility complex, class II, DQ alpha 1 | HLA-DQA1 |  | DOWN |  | UP |  | UP |
| major histocompatibility complex, class II, DQ beta 1 /// major histocompatibility complex, class II, DQ beta 1 | HLA-DQB1 |  |  | UP | UP | UP | UP |
| major histocompatibility complex, class II, DR alpha | HLA-DRA |  |  |  | UP |  | UP |
| major histocompatibility complex, class II, DR beta 1 /// major histocompatibility complex, class II, DR beta 1 /// hypothetical protein LOC730415 /// hypothetical protein LOC730415 | HLA-DRB1 /// LOC730415 |  |  |  | UP | UP | UP |
| heme oxygenase (decycling) 1 | HMOX1 |  |  | UP |  |  |  |
| heat shock protein 90kDa beta (Grp94), member 1 | HSP90B1 |  | DOWN |  | UP |  |  |
| heat shock 70kDa protein 5 (glucose-regulated protein, 78kDa) | HSPA5 |  |  | UP |  |  |  |
| heat shock 70kDa protein 6 (HSP70B') | HSPA6 |  |  |  | UP |  | DOWN |
| heat shock 27kDa protein 1 /// Meis1, myeloid ecotropic viral integration site 1 homolog 3 (mouse) | HSPB1 /// MEIS3 |  |  |  | DOWN |  |  |
| intercellular adhesion molecule 1 (CD54), human rhinovirus receptor | ICAM1 |  |  | UP | UP | UP |  |
| inhibitor of DNA binding 1, dominant negative helix-loop-helix protein | ID1 |  |  | UP | UP | DOWN | UP |
| immediate early response 3 | IER3 |  |  | UP | UP |  |  |
| interferon, gamma-inducible protein 16 | IFI16 |  |  | UP |  |  |  |
| interferon, gamma-inducible protein 30 | IFI30 |  |  |  |  | UP |  |
| interferon-induced protein with tetratricopeptide repeats 1 /// interferon-induced protein with tetratricopeptide repeats 1 | IFIT1 |  |  | DOWN | DOWN |  |  |
| interferon induced transmembrane protein 1 (9-27) | IFITM1 |  |  |  | UP |  |  |
| interferon induced transmembrane protein 2 (1-8D) | IFITM2 |  |  |  | UP |  |  |
| interferon induced transmembrane protein 3 (1-8U) | IFITM3 |  |  |  | UP |  |  |
| interferon gamma receptor 1 | IFNGR1 |  |  | UP | UP |  |  |
| interferon gamma receptor 2 (interferon gamma transducer 1) | IFNGR2 |  |  | UP | UP |  |  |
| Insulin-like growth factor 1 receptor | IGF1R |  |  | UP |  |  |  |
| insulin-like growth factor binding protein 3 | IGFBP3 | UP |  | UP | UP |  |  |
| inhibitor of kappa light polypeptide gene enhancer in B-cells, kinase beta | IKBKB |  |  | UP |  |  |  |
| inhibitor of kappa light polypeptide gene enhancer in B-cells, kinase epsilon | IKBKE |  |  | UP |  |  |  |
| interleukin 13 receptor, alpha 1 | IL13RA1 |  |  |  |  | UP |  |
| interleukin 17C | IL17C |  |  | UP |  |  |  |
| interleukin 19 | IL19 |  |  | UP |  |  |  |
| interleukin 1, alpha | IL1A |  |  | UP | UP | DOWN | UP |
| interleukin 1, beta | IL1B |  |  | UP |  |  |  |
| interleukin 1 family, member 9 | IL1F9 |  |  | UP | UP |  |  |
| interleukin 1 receptor-like 1 | IL1RL1 |  |  |  |  | DOWN |  |
| interleukin 1 receptor antagonist | IL1RN | UP | UP | UP | UP |  |  |
| interleukin 32 /// interleukin 32 | IL32 |  |  | UP | UP |  |  |
| interleukin 4 receptor | IL4R |  |  | UP | UP |  |  |
| interleukin 6 (interferon, beta 2) | IL6 |  |  | UP | UP | UP |  |
| interleukin 6 signal transducer (gp130, oncostatin M receptor) | IL6ST | UP |  | UP | UP | UP |  |
| interleukin 7 | IL7 | DOWN |  | DOWN |  |  |  |
| interleukin 8 | IL8 | UP |  | UP | UP |  |  |
| indoleamine-pyrrole 2,3 dioxygenase | INDO |  | UP | UP | UP | UP |  |
| interleukin-1 receptor-associated kinase 2 | IRAK2 |  |  | UP |  |  |  |
| interleukin-1 receptor-associated kinase 3 | IRAK3 |  |  | UP | UP |  |  |
| interferon regulatory factor 1 | IRF1 |  |  | UP |  |  |  |
| interferon stimulated exonuclease gene 20kDa | ISG20 |  |  | UP |  |  |  |
| integrin, alpha 5 (fibronectin receptor, alpha polypeptide) | ITGA5 |  |  | UP |  |  |  |
| integrin, alpha 6 | ITGA6 |  | DOWN |  |  | DOWN | DOWN |
| integrin, alpha V (vitronectin receptor, alpha polypeptide, antigen CD51) | ITGAV |  |  | UP |  |  |  |
| integrin, beta 4 | ITGB4 |  |  | UP |  |  |  |
| integrin, beta 6 | ITGB6 |  | DOWN | UP |  | DOWN |  |
| integrin, beta 8 | ITGB8 |  |  | UP |  |  |  |
| Jagged 1 (Alagille syndrome) | JAG1 |  |  |  |  |  | DOWN |
| KDEL (Lys-Asp-Glu-Leu) endoplasmic reticulum protein retention receptor 3 | KDELR3 |  |  |  | UP |  |  |
| kinase insert domain receptor (a type III receptor tyrosine kinase) | KDR |  |  | UP | UP | DOWN | DOWN |
| Kruppel-like factor 5 (intestinal) | KLF5 |  |  | UP |  | DOWN |  |
| Kruppel-like factor 6 | KLF6 |  | UP |  |  |  |  |
| kallikrein-related peptidase 10 | KLK10 |  |  |  | DOWN | UP | UP |
| kallikrein-related peptidase 11 | KLK11 |  |  |  | DOWN |  | UP |
| kallikrein-related peptidase 12 | KLK12 |  |  |  |  | UP | UP |
| kallikrein-related peptidase 13 | KLK13 |  |  |  |  | UP | UP |
| laminin, alpha 3 | LAMA3 |  |  |  |  | DOWN |  |
| laminin, gamma 2 | LAMC2 |  | DOWN |  |  | DOWN |  |
| lectin, mannose-binding, 1 | LMAN1 | UP |  | UP | UP |  |  |
| mucin MUC5B /// mucin 5B, oligomeric mucus/gel-forming | LOC649768 /// MUC5B |  |  |  | UP | DOWN |  |
| similar to Complement C3 precursor | LOC653879 |  |  |  | UP |  |  |
| similar to Mucin-5AC (Mucin 5 subtype AC, tracheobronchial) (Tracheobronchial mucin) (TBM) (Major airway glycoprotein) | LOC730855 |  |  | UP | UP | DOWN | DOWN |
| lysyl oxidase-like 2 | LOXL2 |  |  |  | UP | DOWN |  |
| lymphocyte antigen 6 complex, locus D | LY6D |  |  | UP |  | DOWN | DOWN |
| lymphocyte antigen 96 | LY96 |  | UP | UP | UP | UP | UP |
| lysozyme (renal amyloidosis) /// riboflavin kinase | LYZ /// RFK |  | DOWN |  |  | DOWN |  |
| v-maf musculoaponeurotic fibrosarcoma oncogene homolog (avian) | MAF |  | UP |  |  | UP | UP |
| metastasis associated lung adenocarcinoma transcript 1 (non-coding RNA) | MALAT1 | DOWN |  |  | DOWN |  | UP |
| mucosa associated lymphoid tissue lymphoma translocation gene 1 | MALT1 |  |  | UP |  | DOWN |  |
| mitogen-activated protein kinase kinase 1 | MAP2K1 |  |  | UP |  |  |  |
| mitogen-activated protein kinase kinase kinase 5 | MAP3K5 |  |  |  | UP |  |  |
| mitogen-activated protein kinase kinase kinase 8 | MAP3K8 |  |  | UP | UP |  |  |
| mitogen-activated protein kinase kinase kinase kinase 4 | MAP4K4 |  |  | UP |  |  |  |
| mitogen-activated protein kinase 6 | MAPK6 |  |  | UP |  |  |  |
| multiple coagulation factor deficiency 2 | MCFD2 |  |  |  | UP |  |  |
| myeloid cell leukemia sequence 1 (BCL2-related) | MCL1 |  |  | UP |  |  |  |
| met proto-oncogene (hepatocyte growth factor receptor) | MET |  |  | UP |  |  |  |
| MHC class I polypeptide-related sequence B | MICB |  |  | UP |  | DOWN |  |
| MAP kinase interacting serine/threonine kinase 2 | MKNK2 |  |  | UP | UP | DOWN |  |
| matrix metallopeptidase 1 (interstitial collagenase) | MMP1 |  | DOWN |  | UP | DOWN |  |
| matrix metallopeptidase 10 (stromelysin 2) | MMP10 |  | DOWN | UP | UP | DOWN | DOWN |
| matrix metallopeptidase 13 (collagenase 3) | MMP13 |  | DOWN | UP | UP | DOWN | DOWN |
| matrix metallopeptidase 14 (membrane-inserted) | MMP14 |  |  |  | UP |  |  |
| matrix metallopeptidase 2 (gelatinase A, 72kDa gelatinase, 72kDa type IV collagenase) | MMP2 |  |  |  |  |  | DOWN |
| matrix metallopeptidase 28 | MMP28 |  |  |  | UP |  | DOWN |
| matrix metallopeptidase 9 (gelatinase B, 92kDa gelatinase, 92kDa type IV collagenase) | MMP9 |  | DOWN | UP |  | DOWN | DOWN |
| mesothelin | MSLN |  |  |  | UP |  | DOWN |
| mucin 5AC, oligomeric mucus/gel-forming /// similar to Mucin-5AC (Mucin 5 subtype AC, tracheobronchial) (Tracheobronchial mucin) (TBM) (Major airway glycoprotein) | MUC5AC /// LOC730855 |  |  | UP | UP | DOWN | DOWN |
| N-ethylmaleimide-sensitive factor attachment protein, alpha | NAPA |  | UP | DOWN |  |  |  |
| neutrophil cytosolic factor 2 (65kDa, chronic granulomatous disease, autosomal 2) | NCF2 |  | UP |  | DOWN | UP | UP |
| NCK adaptor protein 1 | NCK1 |  |  | UP |  |  |  |
| nuclear factor of activated T-cells 5, tonicity-responsive | NFAT5 |  |  |  |  | UP |  |
| nuclear factor, interleukin 3 regulated | NFIL3 |  |  | UP | UP |  |  |
| nuclear factor of kappa light polypeptide gene enhancer in B-cells 2 (p49/p100) | NFKB2 |  |  | UP | UP |  |  |
| nuclear factor of kappa light polypeptide gene enhancer in B-cells inhibitor, alpha | NFKBIA |  |  | UP | UP |  |  |
| nuclear factor of kappa light polypeptide gene enhancer in B-cells inhibitor, zeta | NFKBIZ |  |  | UP | UP |  |  |
| nidogen 1 | NID1 |  | UP |  |  |  | UP |
| NLR family, pyrin domain containing 2 | NLRP2 |  |  | UP |  |  |  |
| nucleolar protein 3 (apoptosis repressor with CARD domain) | NOL3 |  |  |  | UP |  |  |
| nuclear receptor subfamily 4, group A, member 2 | NR4A2 |  |  |  |  |  | DOWN |
| neuropilin 2 | NRP2 |  |  | UP |  |  |  |
| nuclear protein 1 | NUPR1 |  | UP |  |  |  |  |
| optineurin | OPTN |  |  |  |  | UP |  |
| procollagen-proline, 2-oxoglutarate 4-dioxygenase (proline 4-hydroxylase), beta polypeptide | P4HB |  |  |  | UP |  |  |
| pre-B-cell colony enhancing factor 1 | PBEF1 |  | UP | UP | UP |  |  |
| BH-protocadherin (brain-heart) | PCDH7 |  | UP |  |  | UP |  |
| protocadherin alpha 1-13 /// protocadherin alpha subfamily C, 1 AND 2 | PCDHA 1-13 /// PCDHAC 1,2 |  |  |  | DOWN | UP | UP |
| protein disulfide isomerase family A, member 5 | PDIA5 |  |  |  | UP |  |  |
| placental growth factor, vascular endothelial growth factor-related protein | PGF | UP |  | UP | UP |  |  |
| pleckstrin homology-like domain, family A, member 1 | PHLDA1 |  |  | UP | UP | UP |  |
| pleckstrin homology-like domain, family A, member 2 | PHLDA2 |  |  | UP | UP |  |  |
| Polymeric immunoglobulin receptor | PIGR |  |  |  |  | UP DOWN |  |
| plakophilin 1 (ectodermal dysplasia/skin fragility syndrome) | PKP1 |  |  |  |  |  | DOWN |
| phospholipase A2, group IVA (cytosolic, calcium-dependent) | PLA2G4A |  |  | UP |  |  |  |
| plasminogen activator, tissue | PLAT |  | DOWN | UP | UP | DOWN | DOWN |
| plasminogen activator, urokinase | PLAU |  |  | UP | UP |  | UP |
| plasminogen activator, urokinase receptor | PLAUR |  |  | UP | UP | DOWN |  |
| polo-like kinase 2 (Drosophila) | PLK2 |  |  | UP |  |  |  |
| proteolipid protein 2 (colonic epithelium-enriched) | PLP2 |  |  |  |  |  | DOWN |
| polymerase (DNA directed), beta | POLB |  |  | UP |  |  |  |
| POU domain, class 2, associating factor 1 | POU2AF1 |  |  | DOWN | DOWN | UP |  |
| PTPRF interacting protein, binding protein 1 (liprin beta 1) | PPFIBP1 |  |  | UP |  |  |  |
| peptidylprolyl isomerase B (cyclophilin B) | PPIB |  |  |  | UP |  |  |
| protein kinase, AMP-activated, beta 1 non-catalytic subunit | PRKAB1 |  |  | UP |  |  |  |
| protein kinase C, delta | PRKCD |  |  |  | DOWN |  |  |
| protease, serine, 22 | PRSS22 |  |  | UP | UP |  |  |
| protease, serine, 23 | PRSS23 |  | DOWN |  |  |  |  |
| protease, serine, 3 (mesotrypsin) | PRSS3 |  |  |  |  | DOWN |  |
| prostaglandin E receptor 3 (subtype EP3) | PTGER3 |  | DOWN |  |  |  |  |
| prostaglandin E receptor 4 (subtype EP4) | PTGER4 |  |  | DOWN | DOWN |  |  |
| prostaglandin E synthase | PTGES |  |  | UP | UP |  |  |
| prostaglandin-endoperoxide synthase 2 (prostaglandin G/H synthase and cyclooxygenase) | PTGS2 |  | DOWN | UP | UP |  |  |
| protein tyrosine phosphatase, non-receptor type 11 (Noonan syndrome 1) | PTPN11 |  |  | UP |  |  |  |
| poliovirus receptor-related 2 (herpesvirus entry mediator B) | PVRL2 |  |  | UP |  |  |  |
| poliovirus receptor-related 3 | PVRL3 |  |  |  |  |  | DOWN |
| peroxidasin homolog (Drosophila) | PXDN |  |  |  | UP |  |  |
| RAB31, member RAS oncogene family | RAB31 |  |  | UP | UP |  |  |
| RAB37, member RAS oncogene family | RAB37 |  | DOWN |  |  |  |  |
| RAB7, member RAS oncogene family-like 1 | RAB7L1 |  | UP |  |  | UP | UP |
| RAB8B, member RAS oncogene family | RAB8B |  |  | UP |  |  | UP |
| ras-related C3 botulinum toxin substrate 2 (rho family, small GTP binding protein Rac2) | RAC2 |  |  |  | UP | UP |  |
| v-rel reticuloendotheliosis viral oncogene homolog (avian) | REL |  |  | UP |  |  |  |
| v-rel reticuloendotheliosis viral oncogene homolog A, nuclear factor of kappa light polypeptide gene enhancer in B-cells 3, p65 (avian) | RELA |  |  | UP |  |  |  |
| ring finger and FYVE-like domain containing 1 | RFFL |  |  |  | UP |  |  |
| ras homolog gene family, member B | RHOB |  |  |  | UP |  |  |
| ring finger protein 128 | RNF128 |  |  |  |  | UP |  |
| ropporin, rhophilin associated protein 1B | ROPN1B |  |  |  |  | DOWN | DOWN |
| Ras-related GTP binding C | RRAGC |  | UP |  |  | UP | UP |
| ribosome binding protein 1 homolog 180kDa (dog) | RRBP1 |  |  |  | UP |  |  |
| reticulon 4 | RTN4 |  |  |  |  | UP |  |
| S100 calcium binding protein A12 | S100A12 |  |  | UP | UP | DOWN |  |
| S100 calcium binding protein A7 | S100A7 |  |  | UP |  |  | UP |
| S100 calcium binding protein A8 | S100A8 |  |  |  | UP | DOWN |  |
| S100 calcium binding protein A9 | S100A9 |  |  |  | UP |  |  |
| serum amyloid A1 | SAA1 |  | DOWN | UP | UP |  |  |
| serum amyloid A1 /// serum amyloid A1 /// serum amyloid A2 /// serum amyloid A2 | SAA1 /// SAA2 |  | DOWN | UP | UP |  | UP |
| serum amyloid A4, constitutive | SAA4 |  |  | UP | UP |  |  |
| SAR1 gene homolog A (S. cerevisiae) | SAR1A |  |  |  | DOWN |  | UP |
| secretory carrier membrane protein 1 | SCAMP1 |  |  | UP |  |  |  |
| scavenger receptor class B, member 1 | SCARB1 |  |  |  | UP |  |  |
| secretoglobin, family 3A, member 1 | SCGB3A1 |  |  |  |  | DOWN |  |
| SEC11 homolog C (S. cerevisiae) | SEC11C |  |  |  | UP |  |  |
| SEC24 related gene family, member A (S. cerevisiae) | SEC24A |  |  | UP | UP |  |  |
| SEC24 related gene family, member D (S. cerevisiae) | SEC24D |  |  |  | UP |  |  |
| Sec61 alpha 1 subunit (S. cerevisiae) | SEC61A1 |  |  |  | UP |  |  |
| Sec61 gamma subunit | SEC61G |  |  |  | UP |  |  |
| sema domain, immunoglobulin domain (Ig), short basic domain, secreted, (semaphorin) 3C | SEMA3C |  |  | UP |  |  |  |
| sema domain, transmembrane domain (TM), and cytoplasmic domain, (semaphorin) 6A | SEMA6A |  |  | UP | UP |  |  |
| serpin peptidase inhibitor, clade A (alpha-1 antiproteinase, antitrypsin), member 1 | SERPINA1 |  |  |  | UP |  |  |
| serpin peptidase inhibitor, clade A (alpha-1 antiproteinase, antitrypsin), member 3 | SERPINA3 |  |  | UP | UP |  | UP |
| serpin peptidase inhibitor, clade B (ovalbumin), member 1 | SERPINB1 |  |  | UP | UP | DOWN | DOWN |
| serpin peptidase inhibitor, clade B (ovalbumin), member 2 | SERPINB2 |  | DOWN |  |  | DOWN | DOWN |
| serpin peptidase inhibitor, clade B (ovalbumin), member 4 | SERPINB4 |  |  | UP | UP |  |  |
| serpin peptidase inhibitor, clade B (ovalbumin), member 9 | SERPINB9 |  |  | UP | UP |  | DOWN |
| serpin peptidase inhibitor, clade E (nexin, plasminogen activator inhibitor type 1), member 1 | SERPINE1 |  |  | UP | UP | DOWN |  |
| serum/glucocorticoid regulated kinase | SGK |  |  | UP |  |  |  |
| SMAD family member 3 | SMAD3 |  |  | UP |  | DOWN |  |
| synuclein, alpha (non A4 component of amyloid precursor) /// synuclein, alpha (non A4 component of amyloid precursor) | SNCA |  | UP |  |  |  |  |
| suppressor of cytokine signaling 2 | SOCS2 |  |  | UP | UP | UP |  |
| suppressor of cytokine signaling 3 | SOCS3 |  |  | UP | UP |  |  |
| small optic lobes homolog (Drosophila) | SOLH |  |  | UP |  |  |  |
| serine peptidase inhibitor, Kazal type 5 | SPINK5 |  |  |  |  |  | DOWN |
| sparc/osteonectin, cwcv and kazal-like domains proteoglycan (testican) 1 | SPOCK1 |  |  | DOWN |  |  |  |
| spondin 2, extracellular matrix protein | SPON2 |  | UP |  | DOWN |  |  |
| secreted phosphoprotein 1 (osteopontin, bone sialoprotein I, early T-lymphocyte activation 1) | SPP1 |  | UP |  |  |  | UP |
| sequestosome 1 | SQSTM1 |  | UP |  |  | UP | UP |
| statherin | STATH |  |  |  |  |  | DOWN |
| Stanniocalcin 1 | STC1 | UP |  | UP | UP |  | UP |
| synaptotagmin-like 1 | SYTL1 |  |  |  |  |  | DOWN |
| synaptotagmin-like 2 | SYTL2 |  |  |  | DOWN |  |  |
| TAP binding protein (tapasin) | TAPBP |  |  | UP |  |  |  |
| transferrin receptor (p90, CD71) | TFRC |  | UP |  | UP | UP | UP |
| Transforming growth factor, beta 2 | TGFB2 |  | DOWN |  |  |  | DOWN |
| transforming growth factor, beta-induced, 68kDa | TGFBI |  |  |  | UP |  | DOWN |
| transglutaminase 2 (C polypeptide, protein-glutamine-gamma-glutamyltransferase) | TGM2 |  |  | UP | UP |  |  |
| thrombospondin 1 | THBS1 | UP | DOWN | UP |  |  |  |
| toll-like receptor 1 | TLR1 |  |  | DOWN |  |  |  |
| toll-like receptor 2 | TLR2 |  |  | UP | UP |  |  |
| transmembrane protease, serine 11D | TMPRSS11D |  |  | UP |  |  |  |
| transmembrane protease, serine 2 | TMPRSS2 |  |  | UP |  |  |  |
| transmembrane protease, serine 4 | TMPRSS4 |  |  | UP | UP |  |  |
| tenascin C (hexabrachion) | TNC |  | DOWN |  |  |  | DOWN |
| tumor necrosis factor, alpha-induced protein 2 | TNFAIP2 |  |  | UP | UP |  | DOWN |
| tumor necrosis factor, alpha-induced protein 3 | TNFAIP3 |  |  | UP | UP |  |  |
| tumor necrosis factor receptor superfamily, member 10a | TNFRSF10A |  |  | UP | UP |  |  |
| tumor necrosis factor receptor superfamily, member 10b | TNFRSF10B |  |  | UP |  |  |  |
| tumor necrosis factor receptor superfamily, member 10d, decoy with truncated death domain | TNFRSF10D |  |  | UP | UP |  |  |
| tumor necrosis factor receptor superfamily, member 11a, NFKB activator | TNFRSF11A |  |  | UP |  |  |  |
| tumor necrosis factor receptor superfamily, member 12A | TNFRSF12A |  |  |  |  | DOWN |  |
| tumor necrosis factor receptor superfamily, member 19 | TNFRSF19 |  | UP |  |  |  |  |
| tumor necrosis factor receptor superfamily, member 21 | TNFRSF21 |  |  |  |  |  | DOWN |
| tumor necrosis factor receptor superfamily, member 6b, decoy /// regulator of telomere elongation helicase 1 | TNFRSF6B /// RTEL1 |  |  | UP | UP | DOWN |  |
| tumor necrosis factor (ligand) superfamily, member 10 /// tumor necrosis factor (ligand) superfamily, member 10 | TNFSF10 |  |  |  |  |  | DOWN |
| tumor necrosis factor (ligand) superfamily, member 13 /// tumor necrosis factor (ligand) superfamily, member 12-member 13 | TNFSF13 /// TNFSF12-TNFSF13 |  |  | UP | UP |  |  |
| tumor necrosis factor (ligand) superfamily, member 9 | TNFSF9 |  |  |  |  | UP |  |
| TNFAIP3 interacting protein 1 | TNIP1 |  |  | UP |  |  |  |
| TNFAIP3 interacting protein 2 | TNIP2 |  |  | UP |  |  |  |
| TNFAIP3 interacting protein 3 | TNIP3 |  |  | UP |  |  |  |
| topoisomerase (DNA) II alpha 170kDa | TOP2A |  | DOWN |  | DOWN | DOWN | DOWN |
| tripeptidyl peptidase I | TPP1 |  | UP |  |  | UP | UP |
| triggering receptor expressed on myeloid cells 1 | TREM1 |  |  |  |  |  | UP |
| tribbles homolog 1 (Drosophila) | TRIB1 |  |  | UP | UP | DOWN |  |
| tribbles homolog 2 (Drosophila) | TRIB2 |  |  | UP | UP |  |  |
| triple functional domain (PTPRF interacting) | TRIO |  |  | UP |  |  |  |
| thioredoxin domain containing 5 | TXNDC5 |  |  |  |  | DOWN |  |
| ubiquitin D | UBD | DOWN |  | UP | UP |  |  |
| UL16 binding protein 2 | ULBP2 |  |  |  | UP |  |  |
| vascular endothelial growth factor A | VEGFA |  |  | UP | UP |  | UP |

See footnote to Table 3 in main text in the listed treatment comparisons. Genes are considered differentially regulated if z.pv was <0.05 (see Table 1 from Supplemental Data).
