# Supplementary material for: Azithromycin Treatment Alters Gene Expression in Inflammatory, Lipid Metabolism, and Cell Cycle Pathways in Well-Differentiated Human Airway Epithelia
Source: PLoS One. 2009 Jun 5;4(6):e5806. doi: 10.1371/journal.pone.0005806 (PMC2688381; doi:10.1371/journal.pone.0005806)
Supplement: Table S4 — (0.09 MB DOC) [file pone.0005806.s005.doc]

**Supporting Information.**

**Table S4: Genes up-regulated by AZT, SMM, and AZT + SMM that are annotated in the lipid, steroid, cholesterol, or fatty acid metabolic pathways [Gene Ontology (GO) Biological Process Annotation].**

| **Gene Symbol** | **Gene Name** | **GO Biological Function Description** | **AZT24 vs PBS24** | **SMM 24 vs PBS 24** | **AZT72**  **SMM24 vs SMM24** |
| --- | --- | --- | --- | --- | --- |
| AACS | acetoacetyl-CoA synthetase | isoprenoid biosynthesis | X |  |  |
| ABCA1 | ATP-binding cassette, sub-family A (ABC1), member 1 | lipid metabolism; cholesterol metabolism and transport; phospholipid transport | X |  |  |
| ABCG1 | ATP-binding cassette, sub-family G (WHITE), member 1 | lipid transport; cholesterol metabolism and homeostasis | X |  |  |
| ACACA | acetyl-Coenzyme A carboxylase alpha | fatty acid and lipid biosynthesis | X |  | X |
| ACAT2 | acetyl-Coenzyme A acetyltransferase 2 (acetoacetyl Coenzyme A thiolase) | lipid metabolism | X | X |  |
| ACLY | ATP citrate lyase | lipid biosynthesis | X | X |  |
| ACSL1 | acyl-CoA synthetase long-chain family member 1 | lipid and fatty acid metabolism | X |  | X |
| ACSS2 | acyl-CoA synthetase short-chain family member 2 | lipid biosynthesis | X | X | X |
| AKR1C2 | Aldo-keto reductase family 1, member C2 (dihydrodiol dehydrogenase 2; bile acid binding protein; 3-alpha hydroxysteroid dehydrogenase, type III) | steroid and lipid metabolism, cholesterol absorption and homeostasis |  |  | X |
| ALDH1A3 | Aldehyde dehydrogenase 1 family, member A3 | lipid metabolism | X | X |  |
| ALDH3B2 | aldehyde dehydrogenase 3 family, member B2 | lipid metabolism |  |  | X |
| ALOX15B | arachidonate 15-lipoxygenase, type B | lipid and fatty acid metabolism |  | X |  |
| ANGPTL4 | angiopoietin-like 4 | positive regulation of lipid metabolism |  | X |  |
| APOL1 | apolipoprotein L, 1 | cholesterol, steroid, and lipid metabolism; lipid transport |  | X |  |
| AYTL2 | acyltransferase like 2 | phospholipid biosynthesis |  | X |  |
| CYP51A1 | cytochrome P450, family 51, subfamily A, polypeptide 1 | cholesterol, steroid, sterol, and lipid biosynthesis | X | X | X |
| DHCR7 | 7-dehydrocholesterol reductase | cholesterol, sterol, and lipid biosynthesis | X | X | X |
| EBP | emopamil binding protein (sterol isomerase) | cholesterol, steroid, and lipid biosynthesis | X | X |  |
| FADS1 | fatty acid desaturase 1 | lipid metabolism, fatty acid biosynthesis | X | X | X |
| FADS2 | fatty acid desaturase 2 | lipid metabolism, fatty acid biosynthesis | X | X | X |
| FASN | fatty acid synthase | lipid and fatty acid biosynthesis | X | X | X |
| FDFT1 | farnesyl-diphosphate farnesyltransferase 1 | cholesterol, isoprenoid, steroid, sterol, and lipid biosynthesis | X | X |  |
| FDPS | farnesyl diphosphate synthase (farnesyl pyrophosphate synthetase, dimethylallyltranstransferase, geranyltranstransferase) | cholesterol, isoprenoid, steroid, sterol, and lipid biosynthesis | X | X |  |
| HMGCR | 3-hydroxy-3-methylglutaryl-Coenzyme A reductase | cholesterol, steroid, sterol, and lipid biosynthesis | X | X | X |
| HMGCS1 | 3-hydroxy-3-methylglutaryl-Coenzyme A synthase 1 (soluble) | cholesterol, steroid, sterol, and lipid biosynthesis | X | X | X |
| HPGD | hydroxyprostaglandin dehydrogenase 15-(NAD) | lipid and fatty acid metabolism | X |  | X |
| HSD17B2 | hydroxysteroid (17-beta) dehydrogenase 2 | steroid and lipid biosynthesis |  | X |  |
| IDI1 | isopentenyl-diphosphate delta isomerase 1 | cholesterol, isoprenoid, steroid, sterol, and lipid biosynthesis | X | X | X |
| IGFBP6 | insulin-like growth factor binding protein 6 | cholesterol and lipid metabolism |  | X |  |
| LDLR | low density lipoprotein receptor (familial hypercholesterolemia) | lipid transport; lipid, cholesterol and steroid metabolism, cholesterol homeostasis | X | X | X |
| LPIN1 | lipin 1 | lipid metabolism | X |  |  |
| LSS | lanosterol synthase (2,3-oxidosqualene-lanosterol cyclase) | steroid and lipid biosynthesis | X | X | X |
| LSS /// PCM1 | lanosterol synthase (2,3-oxidosqualene-lanosterol cyclase) /// pericentriolar material 1 | steroid and lipid biosynthesis | X |  | X |
| MVD | mevalonate (diphospho) decarboxylase | cholesterol, isoprenoid, steroid, sterol, and lipid biosynthesis | X | X |  |
| MVK | mevalonate kinase (mevalonic aciduria) | cholesterol, iosprenoid, steroid, sterol, and lipid biosynthesis | X |  |  |
| NPC1 | Niemann-Pick disease, type C1 | cholesterol transport and homeostasis | X |  | X |
| NPC2 | Niemann-Pick disease, type C2 | Regulation of isoprenoid metabolism; cholesterol homeostasis | X |  | X |
| NSDHL | NAD(P) dependent steroid dehydrogenase-like | cholesterol, steroid, sterol, and lipid biosynthesis | X |  |  |
| PDGFC | platelet derived growth factor C | lipid metabolism |  | X |  |
| PSAP | prosaposin (variant Gaucher disease and variant metachromatic leukodystrophy) | lipid and sphingolipid metabolism; lipid transport | X |  | X |
| PTGS2 | prostaglandin-endoperoxide synthase 2 (prostaglandin G/H synthase and cyclooxygenase) | lipid biosynthesis |  | X |  |
| SC4MOL | sterol-C4-methyl oxidase-like | fatty acid and steroid metabolism, sterol and lipid biosynthesis | X | X |  |
| SC5DL | sterol-C5-desaturase (ERG3 delta-5-desaturase homolog, fungal)-like | sterol, steroid, and lipid biosynthesis | X |  |  |
| SCARB1 | scavenger receptor class B, member 1 | cholesterol metabolism |  | X |  |
| SCD | stearoyl-CoA desaturase (delta-9-desaturase) /// stearoyl-CoA desaturase (delta-9-desaturase) | fatty acid and lipid biosynthesis | X | X | X |
| SERPINA3 | serpin peptidase inhibitor, clade A (alpha-1 antiproteinase, antitrypsin), member 3 | regulation of lipid metabolism |  | X | X |
| SQLE | squalene epoxidase | sterol biosynthesis | X | X |  |
| SREBP1 | sterol regulatory element binding transcription factor 1 | cholesterol, steroid, and lipid metabolism | X |  | X |
| STARD4 | START domain containing 4, sterol regulated | steroid biosynthesis; lipid transport | X | X | X |
| TPP1 | tripeptidyl peptidase I | lipid metabolism | X |  | X |

GO: Gene Ontology Biological Process Annotation. To be included in this list, the z score p-value was required to be <0.05. The "X" indicates that the gene was up-regulated by the treatment indicated. A blank means that gene expression was not affected at this level of significance.
