# Supplementary material for: Azithromycin Treatment Alters Gene Expression in Inflammatory, Lipid Metabolism, and Cell Cycle Pathways in Well-Differentiated Human Airway Epithelia
Source: PLoS One. 2009 Jun 5;4(6):e5806. doi: 10.1371/journal.pone.0005806 (PMC2688381; doi:10.1371/journal.pone.0005806)
Supplement: Table S5 — (0.11 MB DOC) [file pone.0005806.s006.doc]

**Supporting Information.**

Table S5. AZT and SMM-dependent down-regulation of genes involved in cell cycle/mitosis processes.

| **Gene Symbol** | **Gene Name** | **GO Annotation** | **AZT6**  **vs.**  **PBS6** | **AZT24**  **vs. PBS24** | **SMM24 vs. PBS24** |
| --- | --- | --- | --- | --- | --- |
| 10-SEP | septin 10 | cell cycle; cell division |  |  | X |
| ANLN | anillin, actin binding protein | regulation of exit from mitosis; cell cycle; cell division; cytokinesis |  | X | X |
| ASPM | asp (abnormal spindle) homolog, microcephaly associated (Drosophila) | cell cycle; mitosis; cell division |  | X |  |
| AURKA | aurora kinase A | mitotic cell cycle |  | X |  |
| AURKB | aurora kinase B | cell cycle; mitosis; cell division |  | X | X |
| BIRC5 | baculoviral IAP repeat-containing 5 | G2/M transition of mitotic cell cycle; cell cycle; mitosis; cell division |  | X | X |
| BUB1 | BUB1 budding uninhibited by benzimidazoles 1 homolog (yeast) | cell cycle; mitosisi; cell division; mitotic spindle checkpoint |  | X | X |
| BUB1B | BUB1 budding uninhibited by benzimidazoles 1 homolog beta (yeast) | cell cycle; mitosis; mitotic checkpoint; spindle organization and biogenesis |  | X |  |
| CCNA2 | cyclin A2 | cell cycle; mitosis; mitotic G2 checkpoint; regulation of cyclin-dependent protein kinase activity |  | X | X |
| CCNB1 | cyclin B1 | G2/M transition; cell cycle; mitosis; cell division | X | X | X |
| CCNB2 | Cyclin B2 | regulation of progression through cell cycle; cell cycle; mitosis; cell division |  | X | X |
| CCND1 | cyclin D1 | G1/S transition of mitotic cell cycle; re-entry into mitotic cell cycle; cell cycle; cell division |  | X |  |
| CDC2 | cell division cycle 2, G1 to S and G2 to M | regulation of progression through cell cycle; traversing start control point of mitotic cell cycle; mitotic G2 checkpoint |  | X | X |
| CDC20 | cell division cycle 20 homolog (S. cerevisiae) | regulation of progression through cell cycle; cell cycle; mitosis; cell division |  | X |  |
| CDC20B | cell division cycle 20 homolog B (S. cerevisiae) | cell cylce; mitosis; cell division |  |  | X |
| CDK6 | cyclin-dependent kinase 6 | regulation of progression through cell cycle; G1 phase of mitotic cell cycle; cell cycle; cell proliferation |  | X | X |
| CDKN3 | cyclin-dependent kinase inhibitor 3 | regulation of cyclin-dependent protein kinase activity; G1/S transition of mitotic cell cycle; cell cycle arrest; negative regulation of cell proliferation |  | X | X |
| CENPA | centromere protein A | nucleosome assembly; chromosome organization and biogenesis |  | X | X |
| CENPF | centromere protein F, 350/400ka (mitosin) | G2 phase of mitotic cell cycle; mitosis; mitotic spindle checkpoint; metaphase plate congression; kinetochore assembly; M phase of mitotic cell cycle; chromosome segregation |  | X | X |
| CEP55 | centrosomal protein 55kDa | cell cycle; cell division; mitosis |  | X | X |
| CHES1 | checkpoint suppressor 1 | DNA damage checkpoint; G2 phase of mitotic cell cycle; cell cycle | X |  |  |
| CROCC | ciliary rootlet coiled-coil, rootletin | cell cycle; centrosome organization and biogenesis |  |  | X |
| CTGF | connective tissue growth factor | DNA replication |  | X |  |
| DHFR | dihydrofolate reductase | nucleotide biosynthesis |  | X | X |
| DLG7 | discs, large homolog 7 | M phase of mitotic cell cycle; mitotic chromosome movement towards spindle pole; positive regulation of mitotic metaphase/anaphase transition |  | X |  |
| DUT | deoxyuridine triphosphatase | DNA replication; nucleotide metabolism; dUTP metabolism; nucleobase, nucleoside, nucleotide and nucleic acid metabolism |  | X |  |
| E2F7 | E2F transcription factor 7 | regulation of progression through the cell cycle; |  |  | X |
| EGFL6 | EGF-like-domain, multiple 6 | cell cycle |  |  | X |
| EGFR | epidermal growth factor receptor (erythroblastic leukemia viral (v-erb-b) oncogene homolog, avian) | positive regulation of cell proliferation; positive regulation of progression through cell cycle; cell cycle | X |  |  |
| GAS1 | growth arrest-specific 1 | cell cycle; cell cycle arrest; negative regulation of cell proliferation; negative regulation of S phase of mitotic cell cycle |  | X |  |
| GINS1 | GINS complex subunit 1 (Psf1 homolog) | DNA replication |  | X |  |
| H2AFV | H2A histone family, member V | nucleosome assembly; chromosome organization and biogenesis |  |  | X |
| H2AFX | H2A histone family, member X | DNA damage checkpoint; DNA repair; DNA recombination; nucleosome assembly; chromosome organization and biogenesis; cell cycle |  | X |  |
| HELLS | helicase, lymphoid-specific | cell cycle; mitosis; maintenance of DNA methylation; centric heterochromatin formation; cell division |  | X |  |
| HIST1H2BG | histone cluster 1, H2bg | nucleosome assembly; chromosome organization and biogenesis |  |  |  |
| HMGB2 | high-mobility group box 2 | DNA replication; DNA unwinding during replication; base-excision repair, DNA ligation/ establishment and/or maintenance of chromatin architecture; nucleosome assembly |  | X |  |
| KIF11 | kinesin family member 11 | microtubule-based movement; cell cycle; mitotic spindle organization and biogenesis; mitosis; mitotic centrosome separation; spindle pole body organization and biogenesis; cell division; mitosis |  | X | X |
| KIF23 | kinesin family member 23 | mitotic spindle elongation; microtubule-based movement; cell cycle; mitosis; cell division |  | X |  |
| KIF2C | kinesin family member 2C | mitosis; cell proliferation; micro-tubule-based movement |  | X |  |
| KLK10 | kallikrein-related peptidase 10 | cell cycle; negative regulation of progression through cell cycle |  |  | X |
| KNTC2 | kinetochore associated 2 | mitotic sister chromatid segregation; cell cycle; spindle organization and biogenesis; cell division; cell cycle; mitosis |  | X |  |
| KPNA2 | karyopherin alpha 2 (RAG cohort 1, importin alpha 1) /// karyopherin alpha-2 subunit like | regulation of DNA recombination; M phase specific microtubule process; G2 phase of mitotic cell cycle; |  | X |  |
| MAD2L1 | MAD2 mitotic arrest deficient-like 1 (yeast) | cell cycle; mitosis; mitotic checkpoint; cell division; mitosis |  | X |  |
| MCM6 | minichromosome maintenance complex component 6 | DNA replication; DNA replication initiation; cell cycle |  | X |  |
| MKI67 | antigen identified by monoclonal antibody Ki-67 | regulation of progression through cell cycle; cell cycle; cell proliferation |  | X | X |
| NCAPG | non-SMC condensin I complex, subunit G | cell cycle; mitosis; mitotic chromosome condensation; cell division |  | X | X |
| NEK2 | NIMA (never in mitosis gene a)-related kinase 2 | mitotic sister chromatid segregation; cell cycle; regulation of mitosis; cell division; mitosis |  |  | X |
| NUF2 | NUF2, NDC80 kinetochore complex component, homolog (S. cerevisiae) | cell cycle; chromosome segregation; mitosis; cell division |  | X |  |
| NUSAP1 | nucleolar and spindle associated protein 1 | cytokinesis after mitosis; mitotic chromosome condensation; establishment of mitotic spindle localization; positive regulation of mitosis; mitotic sister chromatid segregation |  | X | X |
| PAFAH1B1 | platelet-activating factor acetylhydrolase, isoform Ib, alpha subunit 45kDa | establishment of mitotic spindle orientation; cell cycle; mitosis; cell division |  | X |  |
| PAK3 | p21 protein (Cdc42/Rac)-activated kinase 3 | cell cycle; spindle organization and biogenesis; mitosis; cyclin catabolism; positive regulation of exit from mitosis; cell division |  | X | X |
| PBK | PDZ binding kinase | mitosis |  | X | X |
| POLH | Polymerase (DNA directed), eta | DNA replication; DNA repair; pyrimidine dimer repair; postreplication repair |  | X |  |
| POU2AF1 | POU class 2 associating factor 1 | negative regulation of progression through mitotic cell cycle; cell cycle arrest; negative regulation of cell proliferation |  |  | X |
| PRC1 | protein regulator of cytokinesis 1 | mitotic spindle elongation; cytokinesis; cell cycle; cell division |  | X | X |
| PTTG1 | pituitary tumor-transforming 1 | DNA metabolism; DNA repair; cell cycle; chromosome segregation; mitosis; chromosome organization and biogenesis; cell division |  | X |  |
| RACGAP1 | Rac GTPase activating protein 1 | cytokinesis; contractile ring formation; cell cycle; cytokinesis, initiation of separation; cell division |  | X |  |
| RAD51AP1 | RAD51 associated protein 1 | double-stranded break repair via homologous recombination; DNA repair; DNA recombination |  | X | X |
| REV3L | REV3-like, catalytic subunit of DNA polymerase zeta (yeast) | DNA replication; DNA-dependent DNA replication; DNA repair |  |  | X |
| RRM2 | ribonucleotide reductase M2 polypeptide | DNA replicaton; deoxyribonucleoside diphosphate metabolism |  | X |  |
| SGOL2 | shugoshin-like 2 (S. pombe) | cell cycle; chromosome segregation; cell division |  | X |  |
| SMC4 | structural maintenance of chromosomes 4 | DNA metabolism; chromosome organization and biogenesis; cell cycle; chromosome segregation; mitosis; chromosome condensation; mitotic sister chromatid segregation |  | X |  |
| STRN3 | striatin, calmodulin binding protein 3 | cell cycle |  | X |  |
| SUPT16H | suppressor of Ty 16 homolog (S. cerevisiae) | DNA replication; DNA repair; nucleosome disassembly |  |  | X |
| TGFB2 | transforming growth factor, beta 2 | positive regulation of progression through cell cycle |  | X |  |
| TK1 | thymidine kinase 1, soluble | nucleobase, nucleoside, nucleotide and nucleic acid metabolism; DNA replication |  | X |  |
| TOP1 | topoisomerase (DNA) I | DNA replication; DNA topological change | X |  |  |
| TOP2A | topoisomerase (DNA) II alpha 170kDa | DNA replication; DNA topological change; DNA ligation; DNA repair; chromosome segregation |  | X | X |
| TPX2 | TPX2, microtubule-associated, homolog (Xenopus laevis) | mitosis; cell proliferation |  | X | X |
| TTK | TTK protein kinase | regulation of progression through cell cycle; mitotic spindle organization and biogenesis; mitotic spindle checkpoint; positive regulation of cell proliferation |  | X |  |
| TUBB | tubulin, beta | mitotic spindle assembly; cell cycle arrest; cell proliferation |  | X |  |
| TYMS | thymidylate synthetase | nucleobase, nucleoside, nucleotide and nucleic acid metabolism; dTMP biosynthesis; DNA replication; deoxyribonucleoside monophosphate biosynthesis; nucleotide biosynthesis |  | X | X |
| UHRF1 | ubiquitin-like with PHD and ring finger domains 1 | DNA repair; cell cycle; cell proliferation |  | X |  |
| ZWINT | ZW10 interactor , kinetochore associated, homolog (Drosophila) | cell cycle; spindle organization and biogenesis; mitosis; cell division |  | X | X |

To be included in this list, the z score p-value was required to be <0.05. The "X" indicates that the gene was down-regulated by the treatment indicated. A blank means that gene expression was not affected at this level of significance.
