# Supplementary material for: Azithromycin Treatment Alters Gene Expression in Inflammatory, Lipid Metabolism, and Cell Cycle Pathways in Well-Differentiated Human Airway Epithelia
Source: PLoS One. 2009 Jun 5;4(6):e5806. doi: 10.1371/journal.pone.0005806 (PMC2688381; doi:10.1371/journal.pone.0005806)
Supplement: Table S6 — (0.53 MB DOC) [file pone.0005806.s007.doc]

# Supporting Information.

# Multi-Gene Ontology Matrix (MGM) Table S6. For all of the treatment comparisons shown, both significantly up-regulated and down-regulated Gene Ontology Classifications are given. The hypergeometric probabilities are given as negative exponents, and they are not indicated if the computed value was > 0.0001 (-4.0). Numbers are the log10 of the probabilities. The number of probesets included in each column of the analysis are given along with the number of genes represented by these probesets (some genes are represented more than once on the Affymetrix arrays). The number of probesets used in each column is based upon a ranking of significant genes according to z-statistic as described elsewhere in the Methods sections. For example, in column 1, the number of probesets used in the analysis = 10 and these represent the top 10 signaling probesets. In column 2, the top 20 signaling probes are used, etc. Hypergeometric probability values < 7.0 and their corresponding Gene Ontology Classification were used to develop Figure 1 in the main text. For Figure 1, only the most significant hypergeometric probability value for a particular Gene Ontology Classification is given.

# Table S6 MGM-1. Hypergeometric probabilities for each of the significant Gene Ontology classifications listed for the top probesets up-regulated by AZT 6 hr versus PBS 6 hr.

| 10 | 20 | 40 | 80 | 160 | 320 | 640 | Number of Probesets |
| --- | --- | --- | --- | --- | --- | --- | --- |
| 9 | 15 | 31 | 69 | 142 | 269 | 541 | Number of Genes |

|  |  |  |  |  |  |  | **Gene Ontology Classification** |
| --- | --- | --- | --- | --- | --- | --- | --- |
|  |  |  |  |  | -5.1 | -5.3 | angiogenesis |
|  |  |  |  |  | -4.6 | -4.2 | blood coagulation |
| -6.2 | -5.8 | -8.3 | -7.2 | -6.3 | -5.4 | -4.5 | cAMP metabolism |
|  |  | -4.5 | -5.6 | -7.7 | -5.6 | -6.1 | cell-cell signaling |
|  | -4.2 | -4.6 |  |  | -4.3 | -6.1 | epidermis development |
|  |  |  |  |  |  | -5.5 | glycolysis |
| -5.5 | -5.0 | -7.0 | -5.9 | -5.0 | -4.1 |  | lactation |
|  |  | -4.6 |  |  |  |  | neurotransmitter uptake |
|  | -4.3 | -4.8 |  |  |  |  | positive regulation of cell proliferation |
| -6.2 | -5.4 | -6.3 | -4.9 |  |  |  | pregnancy |
|  |  |  |  |  |  | -4.7 | GenMAPP-Glycolysis_and_Gluconeogenesis |
|  |  |  |  |  |  | -4.2 | GenMAPP-Matrix-Metalloproteinases |
|  |  |  |  |  | -4.3 | -6.1 | KEGG-Glycolysis / Gluconeogenesis |
|  |  | -5.9 | -5.2 | -4.6 | -4.0 |  | creatine:sodium symporter activity |
| -5.5 | -4.8 | -5.5 | -6.9 | -5.1 |  |  | hormone activity |
|  |  |  |  |  | -4.3 | -5.0 | plasminogen activator activity |
|  |  |  |  |  |  | -5.9 | protein binding |
|  |  |  |  |  |  | -5.7 | Golgi apparatus |
|  |  |  |  | -4.4 | -5.1 | -4.7 | cytoplasm |
|  |  |  |  |  |  | -4.5 | endoplasmic reticulum |
|  |  |  |  |  |  | -6.7 | extracellular matrix (sensu Metazoa) |
|  |  | -4.4 | -5.6 | -4.9 | -6.5 | -7.0 | extracellular space |
|  |  |  |  |  |  | -4.8 | microsome |

# The numbers of probesets and number of genes tested in each column are listed in the top two rows. See accompanying text for more details concerning the table presentation. Gene Ontology groups not found to be significant for any number of tested genes are not listed.

# Table S6 MGM-2. Hypergeometric probabilities for each of the significant Gene Ontology classifications listed for the top probesets down-regulated by AZT 6 hr versus PBS 6 hr.

| 10 | 20 | 40 | 80 | 160 | 320 | 640 | Number of Probesets |
| --- | --- | --- | --- | --- | --- | --- | --- |
| 10 | 18 | 36 | 73 | 137 | 277 | 559 | Number of Genes |

|  |  |  |  |  |  |  | **Gene Ontology Classification** |
| --- | --- | --- | --- | --- | --- | --- | --- |
|  |  |  |  |  |  |  | NONE SIGNIFICANT |

# The numbers of probesets and number of genes tested in each column are listed in the top two rows. See accompanying text for more details concerning the table presentation. Gene Ontology groups not found to be significant for any number of tested genes are not listed.

# Table S6 MGM-3. Hypergeometric probabilities for each of the significant Gene Ontology classifications listed for the top probesets up-regulated by AZT 24 hr versus PBS 24 hr.

| 10 | 20 | 40 | 80 | 160 | 320 | 640 | Number of Probesets |
| --- | --- | --- | --- | --- | --- | --- | --- |
| 6 | 14 | 28 | 61 | 119 | 243 | 496 | Number of Genes |

|  |  |  |  |  |  |  | **Gene Ontology Classification** |
| --- | --- | --- | --- | --- | --- | --- | --- |
|  |  |  | -6.2 | -5.4 | -4.4 |  | antibacterial humoral response (sensu Vertebrata) |
|  |  |  | -4.3 |  |  |  | antifungal humoral response (sensu Vertebrata) |
|  |  |  |  |  |  | -4.4 | biosynthesis |
|  |  |  | -4.3 |  |  |  | carbohydrate mediated signaling |
|  |  |  |  |  | -4.3 | -5.8 | carbohydrate metabolism |
|  |  | -10.4 | -Inf | -10.3 | -10.8 | -10.4 | cholesterol biosynthesis |
|  |  |  |  |  | -4.1 |  | choline transport |
|  |  |  | -4.8 | -4.3 |  |  | defense response to pathogenic protozoa |
| -4.6 | -8.6 | -9.5 | -9.8 | -8.0 | -6.2 | -6.7 | fatty acid biosynthesis |
|  |  | -5.3 | -4.3 |  |  |  | fatty acid desaturation |
|  |  |  |  | -4.0 |  |  | iron ion homeostasis |
|  |  |  |  |  | -4.1 |  | iron ion transport |
|  |  | -4.0 | -5.4 | -10.1 | -8.9 | -7.0 | isoprenoid biosynthesis |
|  |  |  |  | -4.6 | -5.8 | -7.6 | lipid metabolism |
|  | -4.1 |  |  | -5.8 | -9.2 | -7.6 | metabolism |
|  |  |  | -4.3 |  |  |  | phagocytosis, recognition |
|  |  |  | -4.8 | -4.3 |  |  | reduction of virulence |
|  |  |  | -4.7 |  | -4.3 |  | steroid biosynthesis |
|  |  |  |  |  | -7.1 | -5.8 | sterol biosynthesis |
|  | -4.3 | -10.8 | -Inf | -10.1 | -10.1 | -10.5 | GenMAPP-Cholesterol_Biosynthesis |
| -5.1 | -6.9 | -6.0 | -7.0 | -7.7 | -7.8 | -6.0 | GenMAPP-Fatty_Acid_Synthesis |
|  |  | -6.4 | -7.5 | -10.5 | -10.4 | -10.3 | KEGG-Biosynthesis of steroids |
|  |  |  |  |  |  | -4.2 | KEGG-Carbon fixation |
|  |  |  | -4.3 |  |  |  | KEGG-Fatty acid biosynthesis (path 1) |
|  |  |  |  |  |  | -4.3 | KEGG-Pentose phosphate pathway |
|  |  | -4.6 | -6.2 | -5.4 | -6.4 | -5.2 | KEGG-Terpenoid biosynthesis |
|  |  |  |  |  | -4.1 |  | C-5 sterol desaturase activity |
|  |  |  |  | -4.3 |  |  | benzodiazepine receptor binding |
|  |  |  |  |  | -5.3 | -4.0 | ferric iron binding |
|  |  |  |  |  |  | -4.6 | interleukin-6 receptor activity |
| -4.0 |  |  |  |  |  |  | iron ion binding |
|  |  |  |  |  | -4.1 |  | lathosterol oxidase activity |
|  | -5.4 | -4.9 | -4.7 | -6.3 | -Inf | -9.9 | oxidoreductase activity |
|  |  | -5.0 | -4.3 |  |  |  | oxidoreductase activity, acting on paired donors |
|  |  |  | -4.1 |  |  |  | pattern recognition receptor activity |
| -6.6 | -5.8 | -5.2 | -4.5 |  |  |  | stearoyl-CoA 9-desaturase activity |
|  |  |  | -4.8 | -4.3 |  |  | sterol transporter activity |
|  |  |  |  |  | -6.4 | -4.5 | endoplasmic reticulum |
|  |  |  |  |  | -4.1 |  | ferritin complex |
|  |  |  | -4.0 | -4.7 | -4.8 | -4.3 | integral to membrane |
|  |  |  |  |  | -7.1 | -7.7 | lysosome |

# The numbers of probesets and number of genes tested in each column are listed in the top two rows. See accompanying text for more details concerning the table presentation. Gene Ontology groups not found to be significant for any number of tested genes are not listed.

# Table S6 MGM-4. Hypergeometric probabilities for each of the significant Gene Ontology classifications listed for the top probesets down-regulated by AZT 24 hr versus PBS 24 hr.

| 10 | 20 | 40 | 80 | 160 | 320 | 640 | Number of Probesets |
| --- | --- | --- | --- | --- | --- | --- | --- |
| 10 | 18 | 38 | 69 | 128 | 245 | 484 | Number of Genes |

|  |  |  |  |  |  |  | **Gene Ontology Classification** |
| --- | --- | --- | --- | --- | --- | --- | --- |
|  |  |  |  |  | -5.0 | -5.8 | DNA metabolism |
|  |  |  |  |  | -4.9 |  | G2 phase of mitotic cell cycle |
|  |  |  |  |  | -4.1 |  | M phase specific microtubule process |
|  |  |  | -7.2 | -6.4 | -5.6 | -4.7 | cAMP metabolism |
|  |  |  |  |  | -5.1 |  | cell adhesion |
|  |  |  |  | -6.0 | -6.9 | -7.8 | cell cycle |
|  | -4.1 | -5.5 | -6.7 | -5.6 | -4.5 |  | collagen catabolism |
|  |  | -5.6 | -5.6 | -7.3 | -9.9 | -9.4 | cytokinesis |
|  |  |  |  | -4.7 |  |  | ecotoderm development |
|  |  |  | -4.6 | -7.6 | -5.6 | -7.6 | epidermis development |
|  |  |  | -5.9 | -5.1 | -4.2 |  | lactation |
|  |  |  |  |  |  | -6.6 | microtubule polymerization |
|  |  |  |  | -5.0 | -7.4 | -9.3 | microtubule-based movement |
|  |  | -6.4 | -Inf | -11.4 | -Inf | -Inf | mitosis |
|  |  |  |  | -4.2 |  |  | mitotic spindle elonagation |
|  |  |  | -5.5 |  |  |  | negative regulation of cell proliferation |
|  |  |  |  |  | -4.2 | -5.8 | regulation of cell cycle |
|  |  |  | -5.2 | -8.8 | -8.6 | -8.4 | GenMAPP_Cell_Cycle |
|  |  | -4.8 | -5.7 | -4.7 |  |  | GenMAPP-Matrix_Metalloproteinases |
|  |  |  |  |  | -6.6 | -6.4 | ATP binding |
|  |  |  | -4.4 |  |  |  | collagenase activity |
|  |  |  |  | -4.3 |  |  | heparin binding |
|  |  |  |  | -5.2 | -4.6 |  | microtubule motor activity |
|  |  |  |  |  |  | -4.2 | protein binding |
|  |  |  |  | -6.8 | -6.7 | -6.8 | structural molecule activity |
|  |  |  |  | -4.0 | -4.7 |  | chromosome, pericentric region |
|  |  |  |  |  | -4.0 |  | cytoplasm |
|  |  | -6.1 | -5.7 | -6.8 | -7.6 | -6.0 | extracellular matrix (sensu Metazoa) |
|  |  |  | -5.6 | -4.5 | -4.9 | -4.4 | extracellular space |
|  |  |  |  | -5.6 | -4.0 | -4.0 | intermediate filament |
|  |  |  |  | -4.1 |  |  | kinesin complex |
|  |  |  |  | -5.5 | -4.4 | -4.5 | kinetochore |
|  |  |  |  | -9.9 | -10.3 | -9.9 | spindle |
|  |  |  | -4.2 | -6.0 | -5.2 | -4.3 | spindle microtubule |
|  |  |  |  |  |  | -6.6 | tubulin |

# The numbers of probesets and number of genes tested in each column are listed in the top two rows. See accompanying text for more details concerning the table presentation. Gene Ontology groups not found to be significant for any number of tested genes are not listed.

# Table S6 MGM-5. Hypergeometric probabilities for each of the significant Gene Ontology classifications listed for the top probesets up-regulated by SMM 6 hr versus PBS 6 hr.

| 10 | 20 | 40 | 80 | 160 | 320 | 640 | Number of Probesets |
| --- | --- | --- | --- | --- | --- | --- | --- |
| 8 | 18 | 35 | 67 | 131 | 248 | 485 | Number of Genes |

|  |  |  |  |  |  |  | **Gene Ontology Classification** |
| --- | --- | --- | --- | --- | --- | --- | --- |
|  |  |  |  | -4.4 |  |  | G-protein coupled receptor protein signaling pathway |
|  |  |  |  |  | -5.2 | -4.9 | acute-phase response |
|  |  |  |  | -5.4 | -4.1 | -4.6 | angiogenesis |
|  |  |  | -6.6 | -6.1 | -5.3 | -8.2 | anti-apoptosis |
|  |  |  | -5.6 | -4.2 | -4.8 | -4.6 | blood coagulation |
|  |  |  |  |  |  | -4.7 | cAMP metabolism |
|  |  |  |  |  |  | -4.7 | cell motility |
|  |  |  |  |  |  | -5.3 | cell proliferation |
|  |  |  | -4.2 | -5.7 | -7.2 | -6.3 | cell surface receptor linked signal transduction |
| -7.7 | -5.6 | -6.6 | -9.1 | -Inf | -7.6 | -9.7 | cell-cell signaling |
| -10.2 | -Inf | -9.5 | -11.6 | -Inf | -10.4 | -8.1 | chemotaxis |
|  |  | -5.8 | -5.2 | -4.6 | -4.1 |  | copulation |
|  |  |  | -4.2 |  |  |  | cytoplasmic sequestering of NF-kappaB |
|  |  |  |  |  | -4.5 | -6.6 | epidermis development |
|  |  |  |  |  |  | -4.3 | glutamine biosynthesis |
|  |  |  |  |  |  | -6.3 | immune response |
|  |  |  |  |  |  | -4.5 | induction of apoptosis via death domain receptors |
| -6.7 | -8.6 | -8.3 | -Inf | -9.7 | -6.5 | -7.7 | inflammatory response |
|  |  |  | -4.3 |  |  | -6.0 | negative regulation of cell proliferation |
|  |  |  |  |  | -7.0 | -5.5 | nitrogen compound metabolism |
|  |  |  |  |  |  | -4.9 | positive regulation of I-kappaB kinase/NF-kappaB cascade |
|  |  | -4.5 | -6.0 | -8.9 | -6.3 | -7.6 | positive regulation of cell proliferation |
|  |  |  |  |  |  | -4.8 | protein amino acid phosphorylation |
|  |  |  |  |  |  | -5.3 | regulation of apoptosis |
|  |  |  |  | -4.5 |  | -6.7 | signal transduction |
|  |  |  | -4.8 | -4.2 |  |  | tryptophan catabolism |
|  |  |  |  |  |  | -5.5 | GenMAPP-Apoptosis |
|  |  |  |  |  |  | -5.3 | GenMAPP-Apoptosis_GenMAPP |
|  |  |  | -4.5 |  |  |  | GenMAPP-Blood_Clotting_Cascade |
|  |  |  |  |  |  | -4.4 | GenMAPP-Hypertrophy_model |
|  |  |  |  | -4.2 |  |  | GenMAPP-Wnt_signaling |
|  |  |  |  |  |  | -5.1 | ATP binding |
|  |  |  | -5.2 | -4.6 | -4.1 |  | U-plasminogen activator receptor activity |
| -10.2 | -Inf | -11.9 | -10.7 | -8.5 | -6.6 | -4.7 | chemokine activity |
|  |  |  |  | -4.6 | -4.1 |  | creatine:sodium symporter activity |
|  |  | -5.0 |  | -5.0 |  | -4.0 | cytokine activity |
|  |  |  |  |  |  | -4.3 | glutamate-ammonia ligase activity |
|  |  |  |  |  | -4.2 |  | hydrolase activity, acting on carbon-nitrogen (but not peptide) bonds |
|  |  |  |  |  |  | -4.7 | interferon-gamma receptor activity |
|  |  | -5.3 | -4.8 | -4.2 |  |  | interleukin-6 receptor binding |
|  |  |  |  |  | -4.2 |  | lipid transporter activity |
|  |  |  |  |  | -5.5 | -4.7 | oncostatin-M receptor activity |
|  |  |  | -6.1 | -5.2 | -4.4 | -5.2 | plamsinogen activator activity |
|  |  |  |  |  |  | -8.3 | protein binding |
|  |  | -5.2 | -5.5 | -5.2 | -5.7 | -5.2 | serine-type endopeptidase inhibitor activity |
|  |  |  |  |  |  | -4.7 | signal transducer activity |
|  |  |  |  |  |  | -4.4 | cytoplasm |
| -4.9 | -5.9 | -6.2 | -4.0 | -5.3 | -7.0 | -6.6 | extracellular region |
| -5.8 | -7.2 | -6.7 | -10.9 | -11.2 | -10.2 | -Inf | extracellular space |
|  |  |  |  |  | -4.1 |  | oncostatin-M receptor complex |

# The numbers of probesets and number of genes tested in each column are listed in the top two rows. See accompanying text for more details concerning the table presentation. Gene Ontology groups not found to be significant for any number of tested genes are not listed.

# Table S6 MGM-6. Hypergeometric probabilities for each of the significant Gene Ontology classifications listed for the top probesets down-regulated by SMM 6 hr versus PBS 6 hr.

| 10 | 20 | 40 | 80 | 160 | 320 | 640 | Number of Probesets |
| --- | --- | --- | --- | --- | --- | --- | --- |
| 9 | 18 | 36 | 72 | 144 | 275 | 533 | Number of Genes |

|  |  |  |  |  |  |  | **Gene Ontology Classification** |
| --- | --- | --- | --- | --- | --- | --- | --- |
|  |  |  |  |  |  |  | NONE SIGNIFICANT |

# The numbers of probesets and number of genes tested in each column are listed in the top two rows. See accompanying text for more details concerning the table presentation. Gene Ontology groups not found to be significant for any number of tested genes are not listed.

# Table S6 MGM-7. Hypergeometric probabilities for each of the significant Gene Ontology classifications listed for the top probesets up-regulated by SMM 24 hr versus PBS 24 hr.

| 10 | 20 | 40 | 80 | 160 | 320 | 640 | Number of Probesets |
| --- | --- | --- | --- | --- | --- | --- | --- |
| 10 | 19 | 36 | 67 | 129 | 251 | 477 | Number of Genes |

|  |  |  |  |  |  |  | **Gene Ontology Classification** |
| --- | --- | --- | --- | --- | --- | --- | --- |
| -4.3 | -8.4 | -9.4 | -8.0 | -6.6 | -6.6 | -5.0 | acute-phase response |
|  |  |  |  |  | -4.1 |  | angiogenesis |
|  |  |  |  |  |  | -4.0 | anti-apoptosis |
|  |  |  |  |  | -7.9 | -10.5 | antigen presentation, exogenous antigen |
|  |  |  |  |  | -7.7 | -10.3 | antigen processing, exogenous antigen via MHC class II |
|  |  |  |  |  | -4.8 | -6.4 | blood coagulation |
|  |  |  |  |  | -4.5 | -6.4 | cell surface receptor linked signal transduction |
|  |  | -4.0 | -6.7 | -6.9 | -6.7 | -7.8 | cell-cell signaling |
| -4.9 | -7.6 | -7.8 | -9.0 | -9.3 | -9.7 | -8.2 | chemotaxis |
|  |  |  |  | -7.0 | -Inf | -9.7 | cholesterol biosynthesis |
| -6.9 | -6.3 | -5.8 | -5.2 | -4.7 | -4.1 |  | copulation |
|  |  |  |  |  | -4.5 | -6.6 | epidermis development |
|  |  |  |  |  | -8.2 | -10.1 | immune response |
| -4.4 | -5.0 | -6.6 | -8.9 | -8.5 | -6.5 | -7.1 | inflammatory response |
|  |  |  |  |  | -5.2 | -4.1 | isoprenoid biosynthesis |
|  |  | -4.2 |  |  |  |  | keratinocyte differentiation |
|  |  |  | -4.3 |  |  |  | negative regulation of cell proliferation |
|  | -5.0 | -4.5 |  |  |  |  | neutrophil chemotaxis |
|  |  |  |  | -4.2 |  |  | positive regulation of cell adhesion |
|  |  |  |  |  |  | -4.0 | proteolysis and peptidolysis |
|  |  |  |  |  | -4.4 |  | response to biotic stimulus |
|  |  |  |  | -7.3 | -Inf | -10.5 | GenMAPP-Cholesterol_Biosynthesis |
|  |  |  |  |  | -4.1 |  | GenMAPP-Complement_Activation_Classical |
|  |  |  |  |  | -5.1 | -8.6 | KEGG-Biosynthesis of steroids |
|  |  |  |  |  | -4.4 | -5.2 | KEGG-Terpenoid biosynthesis |
|  |  |  |  |  | -7.3 | -9.9 | MHC class II receptor activity |
|  |  |  |  | -4.1 |  |  | U-plasminogen activator receptor activity |
|  | -7.5 | -8.3 | -8.7 | -8.6 | -6.6 | -4.7 | chemokine activity |
|  |  |  |  |  | -4.1 |  | creatine:sodium symporter activity |
|  |  |  |  |  |  | -4.9 | electron transporter activity |
|  |  |  |  |  |  | -4.7 | interferon-gamma receptor activity |
|  |  | -5.3 | -4.8 | -4.2 |  |  | interleukin-6 receptor binding |
|  |  |  |  |  | -4.9 | -6.8 | isomerase activity |
|  | -5.4 | -4.5 |  | -4.2 | -4.2 |  | lipid transporter activity |
|  |  |  |  |  |  | -4.7 | oncostatin-M receptor activity |
|  |  |  |  | -4.4 | -10.4 | -Inf | oxidoreductase activity |
|  |  |  |  |  | -4.4 |  | plasminogen activator activity |
|  |  |  |  |  | -4.4 |  | protein-lysine 6 oxidase activity |
| -5.4 | -4.5 | -6.9 | -5.5 | -6.5 | -6.7 | -9.1 | serine-type endopeptidase inhibitor activity |
|  |  |  |  |  | -4.8 | -10.3 | endoplasmic reticulum |
|  |  |  |  |  |  | -4.7 | endoplasmic reticulum membrane |
| -4.4 | -8.6 | -8.5 | -7.8 | -10.7 | -Inf | -Inf | extracellular region |
|  |  | -5.3 | -9.2 | -10 | -Inf | -10.5 | extracellular space |
|  |  |  |  |  | -7.0 | -9.7 | integral to membrane |
|  |  |  |  |  |  | -7.2 | integral to plasma membrane |
|  |  |  |  |  |  | -5.5 | membrane |
|  |  |  |  |  | -4.1 |  | oncostatin-M receptor complex |
|  |  |  | -4.6 | -4.8 | -4.2 |  | soluble fraction |

# The numbers of probesets and number of genes tested in each column are listed in the top two rows. See accompanying text for more details concerning the table presentation. Gene Ontology groups not found to be significant for any number of tested genes are not listed.

# Table S6 MGM-8. Hypergeometric probabilities for each of the significant Gene Ontology classifications listed for the top probesets down-regulated by SMM 24 hr versus PBS 24 hr.

| 10 | 20 | 40 | 80 | 160 | 320 | 640 | Number of Probesets |
| --- | --- | --- | --- | --- | --- | --- | --- |
| 9 | 18 | 34 | 65 | 128 | 253 | 505 | Number of Genes |

|  |  |  |  |  |  |  | **Gene Ontology Classification** |
| --- | --- | --- | --- | --- | --- | --- | --- |
|  |  |  |  |  |  | -5.4 | cell cycle |
|  |  |  |  |  | -4.8 | -6.7 | cytokinesis |
|  |  |  |  | -4.7 | -4.6 | -4.1 | electron transport |
|  |  | -5.4 | -4.8 | -4.2 |  |  | hormone biosynthesis |
|  |  |  |  | -5.0 | -8.3 | -10.5 | mitosis |
|  |  | -4.7 | -4.1 |  |  |  | proteoglycan biosynthesis |
|  |  |  |  |  | -4.3 | -6.3 | Gen MAPP-Cell_cycle_KEGG |
|  |  |  |  |  |  | -4.0 | KEGG-Bile acid biosynthesis |
|  |  |  |  |  | -4.5 | -5.5 | KEGG-Fatty acid metabolism |
|  |  |  |  |  | -4.6 | -4.7 | KEGG-tryptophan metabolism |
|  |  | -5.1 | -4.5 |  |  |  | N-acetylgalactosamine 4-O-sulfotransferase activity |
|  |  |  | -4.6 |  | -4.2 |  | monooxygenase activity |
|  |  |  |  |  |  | -4.4 | transferase activity |
|  |  |  |  |  | -6.7 | -6.2 | unspecified monooxygenase activity |
|  |  |  |  |  | -4.2 |  | membrane |
|  |  |  |  | -4.4 | -7.2 | -6.6 | microsome |
|  |  |  |  |  |  | -6.1 | nucleus |
|  |  |  |  |  | -4.9 | -5.1 | spindle |
|  |  |  |  |  | -5.1 | -4.2 | spindle microtubule |

# The numbers of probesets and number of genes tested in each column are listed in the top two rows. See accompanying text for more details concerning the table presentation. Gene Ontology groups not found to be significant for any number of tested genes are not listed.

# Table S6 MGM-9. Hypergeometric probabilities for each of the significant Gene Ontology classifications listed for the top probesets up-regulated by AZT 48 hr (+ SMM 6 hr) versus SMM 6 hr.

| 10 | 20 | 40 | 80 | 160 | 320 | 640 | Number of Probesets |
| --- | --- | --- | --- | --- | --- | --- | --- |
| 7 | 16 | 30 | 64 | 125 | 241 | 473 | Number of Genes |

|  |  |  |  |  |  |  | **Gene Ontology Classification** |
| --- | --- | --- | --- | --- | --- | --- | --- |
|  |  |  |  |  | -4.4 |  | antibacterial humoral response (sensu Vertebrata) |
|  |  |  | -4.3 |  |  |  | antifungal humoral response (sensu Vertebrata) |
|  |  |  | -4.3 |  |  |  | carbohydrate mediated signaling |
|  |  |  |  |  |  | -4.7 | carbohydrate metabolism |
|  |  |  |  | -7.1 | -Inf | -11.1 | cholesterol biosynthesis |
|  |  |  | -4.8 | -4.2 |  |  | defense response to pathogenic protozoa |
| -4.5 | -8.3 | -9.4 | -9.6 | -9.6 | -7.6 | -5.6 | fatty acid biosynthesis |
|  |  | -4.9 | -4.3 |  |  |  | fatty acid desaturation |
|  |  |  |  |  |  | -4.0 | glycosphingolipid metabolism |
|  |  |  | -4.2 |  |  | -4.8 | humoral immune response |
|  |  |  |  |  | -4.1 |  | iron ion transport |
|  |  |  |  |  | -5.3 | -5.6 | isoprenoid biosynthesisis |
|  |  |  |  |  | -5.8 | -7.1 | lipid metabolism |
|  |  |  |  | -4.1 |  | -4.3 | metabolism |
|  |  |  | -4.3 |  |  |  | phagocytosis, recognition |
|  |  |  | -4.8 | -4.2 |  |  | reduction of virulence |
|  |  |  |  |  |  | -4.0 | sterol biosynthesis |
|  |  |  | -6.7 | -10.3 | -Inf | -10.8 | GenMAPP-Cholesterol_Biosynthesis |
| -5.0 | -6.7 | -5.9 | -4.9 | -5.7 | -4.6 | -6.1 | GenMAPP-Fatty_Acid_Synthesis |
|  |  |  |  | -4.4 | -6.9 | -8.6 | KEGG-Biosynthesis of Steroids |
|  |  |  |  |  | -4.4 |  | KEGG-Terpenoid biosynthesis |
|  |  |  | -4.8 | -4.2 |  |  | benzodiazepine receptor binding |
|  |  |  |  |  |  | -4.2 | hydrolase activity, acting on glycosyl bonds |
|  |  |  |  | -4.3 |  |  | lipid binding |
|  |  |  |  |  |  | -4.4 | mannosyl-oligosaccharide 1,2-alpha-mannosidase activity |
|  |  |  | -5.5 | -5.3 | -6.7 | -9.8 | oxidoreductase activity |
|  |  | -4.9 | -4.3 |  |  |  | oxidoreductase activity, acting on paired donors |
|  |  |  | -4.1 |  | -4.9 | -4.0 | pattern recognition receptor activity |
| -6.5 | -5.7 | -5.2 | -7.3 | -6.4 | -5.6 | -4.7 | stearoyl-CoA 9-desaturase activity |
|  |  |  |  |  | -4.4 | -7.1 | endoplasmic reticulum |
|  |  |  |  |  | -4.1 |  | ferritin complex |
|  | -4.1 |  |  |  |  | -5.3 | integral to membrane |
|  |  |  |  |  | -7.1 | -7.9 | lysosome |
|  |  |  |  |  | -4.2 |  | peroxisome |

# The numbers of probesets and number of genes tested in each column are listed in the top two rows. See accompanying text for more details concerning the table presentation. Gene Ontology groups not found to be significant for any number of tested genes are not listed.

# Table S6 MGM-10. Hypergeometric probabilities for each of the significant Gene Ontology classifications listed for the top probesets down-regulated by AZT 48 hr (+ SMM 6 hr) versus SMM 6 hr.

| 10 | 20 | 40 | 80 | 160 | 320 | 640 | Number of Probesets |
| --- | --- | --- | --- | --- | --- | --- | --- |
| 8 | 16 | 30 | 62 | 132 | 267 | 533 | Number of Genes |

|  |  |  |  |  |  |  | **Gene Ontology Classification** |
| --- | --- | --- | --- | --- | --- | --- | --- |
|  |  |  |  |  |  | -4.5 | DNA replication |
|  |  |  |  |  | -5.4 | -4.5 | cAMP metabolism |
|  | -6.7 | -5.8 | -4.8 |  | -4.3 |  | collagen catabolism |
|  |  |  |  |  | -5.5 | -8.0 | cytokinesis |
|  |  |  | -9.8 | -8.9 | -10.8 | -9.3 | epidermis development |
|  |  |  |  |  | -4.1 |  | lactation |
|  |  |  |  |  | -5.0 | -5.9 | mitosis |
|  |  |  |  |  | -5.1 | -7.8 | GenMAPP-Cell_Cycle_KEGG |
|  | -6.0 | -5.1 | -4.2 |  |  |  | GenMAPP-Matrix_Metalloproteinases |
|  |  |  |  |  | -4.3 |  | plasminogen activator activity |
|  |  | -4.4 | -9.3 | -7.6 | -8.4 | -5.0 | structural molecule activity |
|  |  |  | -4.9 | -5.7 | -5.5 | -4.3 | extracellular matrix (sensu Metazoa) |
|  |  |  |  |  | -4.5 | -5.4 | extracellular space |
|  |  |  | -7.5 | -5.5 | -5.9 | -5.4 | intermediate filament |

The numbers of probesets and number of genes tested in each column are listed in the top two rows. See accompanying text for more details concerning the table presentation. Gene Ontology groups not found to be significant for any number of tested genes are not listed.

# Table S6 MGM-11. Hypergeometric probabilities for each of the significant Gene Ontology classifications listed for the top probesets up-regulated by AZT 72 hr (+ SMM 24 hr) versus SMM 24 hr.

| 10 | 20 | 40 | 80 | 160 | 320 | 640 | Number of Probesets |
| --- | --- | --- | --- | --- | --- | --- | --- |
| 7 | 15 | 32 | 63 | 127 | 252 | 474 | Number of Genes |

|  |  |  |  |  |  |  | **Gene Ontology Classification** |
| --- | --- | --- | --- | --- | --- | --- | --- |
| -4.6 |  |  | -4.4 | -5.0 |  |  | acute-phase response |
|  |  |  |  |  | -4.4 |  | antibacterial humoral response (sensu Vertebrata) |
|  |  |  |  |  |  | -4.7 | canalicular bile acid transport |
| -5.2 | -4.5 |  | -5.3 | -4.4 |  |  | cell migration |
|  |  |  |  | -4.4 | -6.7 |  | cell-cell signaling |
|  |  |  |  | -5.3 | -7.1 | -8.2 | cholesterol biosynthesis |
|  |  | -5.0 | -4.1 | -6.2 | -6.1 | -4.5 | fatty acid biosynthesis |
|  |  |  |  |  | -4.8 | -4.0 | gycosphingolipid metabolism |
|  |  |  | -4.3 |  | -5.1 | -4.3 | induction of positive chemotaxis |
|  |  |  |  |  |  | -4.1 | iron ion transport |
|  |  |  |  |  | -5.6 | -7.9 | lipid metabolism |
|  |  | -4.7 |  | -5.2 | -7.5 | -9.7 | metabolism |
|  |  |  |  | -4.6 | -4.7 |  | proteolysis and peptidolysis |
| -4.9 | -4.2 |  |  |  |  |  | response to wounding |
|  |  |  |  |  | -5.2 | -5.2 | small GTPase mediated signal transduction |
|  |  |  |  |  |  | -4.0 | sterol biosynthesis |
|  |  |  |  | -9.2 | -Inf | -10.6 | GenMAPP-Cholesterol_Biosynthesis |
|  | -4.3 |  |  |  | -4.5 | -4.7 | GenMAPP-Fatty_Acid_Synthesis |
| -4.4 |  |  |  |  |  |  | GenMAPP-Inflammatory_Response_Pathway |
|  |  |  |  |  | -5.1 | -7.0 | KEGG-Biosynthesis of steroids |
|  |  |  |  |  | -4.4 |  | KEGG-Terpenoid biosynthesis |
|  |  |  |  |  |  | -4.7 | 3-alpha-hyroxysteroid dehydrogenase (A-specific) activity |
|  |  |  |  |  |  | -4.0 | bile acid transporter activity |
| -4.9 | -4.2 |  |  |  |  |  | collagen binding |
|  |  |  |  |  | -5.3 | -4.1 | cytokine activity |
| -5.8 | -4.6 |  |  |  |  |  | extracellular matrix structural constituent |
|  |  |  |  |  |  | -4.1 | ferric iron binding |
|  |  |  | -4.4 |  |  |  | heparin binding |
|  |  |  |  |  |  | -4.7 | interleukin-6 receptor activity |
|  |  |  |  |  |  | -4.6 | magnesium ion binding |
|  |  |  |  | -6.0 | -5.7 | -7.9 | oxidoreductase activity |
|  | -5.8 | -5.1 | -4.5 |  |  |  | stearoyl-CoA 9-desaturase activity |
|  |  | -4.3 | -8.1 | -8.7 | -6.2 | -4.8 | extracellular region |
|  |  |  |  |  | -4.7 |  | extracellular space |
|  |  |  |  |  | -5.4 | -5.3 | integral to membrane |
|  |  |  |  |  | -8.0 | -8.8 | lysosome |
|  |  |  |  |  | -4.4 |  | plasma membrane |

# The numbers of probesets and number of genes tested in each column are listed in the top two rows. See accompanying text for more details concerning the table presentation. Gene Ontology groups not found to be significant for any number of tested genes are not listed.

# Table S6 MGM-12. Hypergeometric probabilities for each of the significant Gene Ontology classifications listed for the top probesets down-regulated by AZT 72 hr (+ SMM 24 hr) versus SMM 24 hr.

| 10 | 20 | 40 | 80 | 160 | 320 | 640 | Number of Probesets |
| --- | --- | --- | --- | --- | --- | --- | --- |
| 10 | 20 | 34 | 69 | 136 | 266 | 537 | Number of Genes |

|  |  |  |  |  |  |  | **Gene Ontology Classification** |
| --- | --- | --- | --- | --- | --- | --- | --- |
|  |  |  |  |  | -4.3 | -4.4 | collagen catabolism |
|  |  |  |  | -4.6 | -4.0 |  | copulation |
|  |  |  | -6.9 | -5.9 | -8.3 | -8.7 | cytokinesis |
|  |  |  |  | -4.6 |  | -4.2 | ectoderm development |
|  |  |  |  | -7.4 | -6.6 | -9.3 | epidermis development |
|  |  |  | -8.0 | -9.9 | -9.2 | -8.6 | mitosis |
|  |  |  |  |  |  | -4.0 | mitotic spindle organization and biogenesis |
|  |  |  |  |  |  | -4.4 | protein biosynthesis |
|  |  |  |  |  |  | -4.1 | regulation of cell cycle |
|  |  |  | -6.6 | -6.1 | -6.1 | -6.0 | GenMAPP-Cell_cycle_KEGG |
|  |  |  | -4.0 | -4.6 | -6.0 | -6.5 | GenMAPP-Matrix_Metalloproteinases |
|  |  |  |  |  |  | -8.5 | GenMAPP-Ribosomal_Proteins |
|  |  |  |  |  |  | -6.0 | ATP binding |
|  |  |  |  |  | -5.4 | -4.2 | cyclin-dependent protein kinase activity |
|  |  |  |  | -5.6 | -4.2 |  | oxygen binding |
|  |  |  |  |  | -4.5 | -4.1 | serine-type endopeptidase inhibitor activity |
|  |  |  |  | -4.3 | -4.4 |  | structural constituent of cytoskeleton |
|  |  |  |  |  |  | -5.2 | structural constituent of ribosome |
|  |  | -4.1 |  |  | -4.8 | -5.5 | structural molecule activity |
|  |  |  |  |  |  | -5.9 | cytosolic large ribosomal subunit (sensu Eukaryota) |
|  |  |  | -4.6 | -8.7 | -8.0 | -7.4 | extracellular matrix (sensu Metazoa) |
|  |  |  |  |  | -5.2 |  | extracellular space |
|  | -4.6 | -5.5 | -5.7 | -8.1 | -8.1 | -6.2 | intermediate filament |
|  |  |  |  |  |  | -4.0 | ribosome |
|  |  |  |  |  | -4.8 | -5.0 | spindle |

The numbers of probesets and number of genes tested in each column are listed in the top two rows. See accompanying text for more details concerning the table presentation. Gene Ontology groups not found to be significant for any number of tested genes are not listed.
